# Implications of Cardiovascular Disease Risk Assessment Using the WHO/ISH Risk Prediction Charts in Rural India

**DOI:** 10.1371/journal.pone.0133618

**Published:** 2015-08-19

**Authors:** Arvind Raghu, Devarsetty Praveen, David Peiris, Lionel Tarassenko, Gari Clifford

**Affiliations:** 1 Institute of Biomedical Engineering, Department of Engineering Science, University of Oxford, Oxford, United Kingdom; 2 The George Institute of Global Health, Hyderabad, India; 3 University of Sydney, Sydney, NSW, Australia; 4 The George Institute of Global Health, Sydney, Australia; 5 Emory University, Atlanta, United States of America; 6 Georgia Institute of Technology, Atlanta, United States of America; National Institutes of Health, UNITED STATES

## Abstract

Cardiovascular disease (CVD) risk in India is currently assessed using the World Health Organization/International Society for Hypertension (WHO/ISH) risk prediction charts since no population-specific models exist. The WHO/ISH risk prediction charts have two versions—one with total cholesterol as a predictor (the high information (HI) model) and the other without (the low information (LI) model). However, information on the WHO/ISH risk prediction charts including guidance on which version to use and when, as well as relative performance of the LI and HI models, is limited. This article aims to, firstly, quantify the relative performance of the LI and HI WHO/ISH risk prediction (for WHO-South East Asian Region D) using data from rural India. Secondly, we propose a pre-screening (simplified) point-of-care (POC) test to identify patients who are likely to benefit from a total cholesterol (TC) test, and subsequently when the LI model is preferential to HI model. Analysis was performed using cross-sectional data from rural Andhra Pradesh collected in 2005 with recorded blood cholesterol measurements (N = 1066). CVD risk was computed using both LI and HI models, and *high risk individuals who needed treatment*(*T*_*HR*_) were subsequently identified based on clinical guidelines. Model development for the POC assessment of a TC test was performed through three machine learning techniques: Support Vector Machine (SVM), Regularised Logistic Regression (RLR), and Random Forests (RF) along with a feature selection process. Disagreement in CVD risk predicted by LI and HI WHO/ISH models was 14.5% (n = 155; p<0.01) overall and comprised 36 clinically relevant *T*_*HR*_ patients (31% of patients identified as *T*_*HR*_ by using either model). Using two patient-specific parameters (age, systolic blood pressure), our POC assessment can pre-determine the benefit of TC testing and choose the appropriate risk model (out-of-sample AUCs:RF-0.85,SVM-0.84,RLR:0.82 and maximum sensitivity-98%). The identification of patients benefitting from a TC test for CVD risk stratification can aid planning for resource-allocation and save costs for large-scale screening programmes.

## Introduction

The prevalence of cardiovascular disease (CVD) is increasing in the developing world [1]. The Indian subcontinent accounts for the highest rates of CVD globally [2]. Although many algorithms for CVD risk assessment have been developed worldwide, no analysis of cohort data is available in these regions for population-specific development of CVD risk models. Currently, the CVD risk prediction charts developed by the World Health Organization (WHO) and International Society for Hypertension (ISH) [3] is the only algorithm in the Indian subcontinent prescribed for CVD risk assessment by respective national guidelines [4]. For instance, in India, interventions for CVD and associated risk factors like diabetes are through the National Programme for Prevention and Control of Cancer, Diabetes, Cardiovascular diseases and Stroke (NPCDCS) that prescribes the WHO/ISH South East Asian Region -D (SEAR-D) charts for CVD risk assessment [5]. SEAR-D countries include Bangladesh, Bhutan, Democratic People’s Republic of Korea, India, Maldives, Myanmar, and Nepal [3].

The WHO/ISH risk prediction charts are a series of colour-coded charts recommended by the WHO guidelines for CVD prevention. Different charts are available for the 14 WHO epidemiological subregions around the world [3]. The WHO/ISH risk prediction charts for SEAR-D are illustrated in the supporting information file to this article (Fig B and Fig C in S1 File). They predict a quantised range of 10-year fatal or non-fatal CVD risk. The charts are available in two versions: the low information model (LI) requires age, gender, systolic blood pressure, smoking status and presence of diabetes mellitus to predict 10-year CVD risk; the high information (HI) model uses all the LI model predictors as well as total cholesterol (TC) for risk prediction. There are five levels of quantisation in the 10-year risk model; less than 10%; 10 to <20%; 20 to <30%; 30 to <40%; and ≥ 40%. In the literature there is limited information about the accuracy or validation procedure of the WHO/ISH risk prediction charts. The charts were not developed using prospective or out-of-sample test data and the methods employed differ from other risk estimation functions [6]. Performance metrics such as the classification error between the LI and HI models have also not been reported for SEAR-D.

In this article, we address two key issues concerning risk prediction with the WHO/ISH risk prediction charts, specifically for the SEAR-D population:

- Objective 1: Quantify the relative error between the LI and HI WHO/ISH risk prediction charts based on data from rural India, specifically highlighting those patients who are likely to be under- or over-treated as a result of choosing one version of the charts over the other.
- Objective 2: Use the same dataset to develop a sparse point-of-care (POC) algorithm which allows the user to identify patients who are likely to benefit from a TC test when their CVD risk is being assessed through the WHO/ISH risk prediction charts.

We make the assumption that the WHO/ISH HI model is more accurate than the LI model because it uses more information (through the inclusion of total cholesterol, a strong predictor of CVD). Given that there are over 3 million new CVD cases a year, we foresee the implications of this study to facilitate more accurate screening of CVD risk in India. Additionally, we envisage that this study will be of practical relevance to healthcare policy makers and payers, especially in low- and middle-income countries, who may not be able to fund TC tests for the whole population.

## Methods

### Data

The Andhra Pradesh Rural Health Initiative (APHRI) is a cross sectional study of CVD risk factors for 4535 subjects (48.6% male) in rural Andhra Pradesh [7]. Participants from over 20 villages were stratified by age and gender and selected by randomly sampling. Each age and gender stratum could therefore be represented equally (especially the elderly participants who are more vulnerable to a CVD event). Blood samples were drawn from every fourth participant in the study. More details on the APHRI study can be found in the article by Chow et al. (2008) [8]. For our analysis, only those subjects who had recorded blood cholesterol and blood glucose measurements were included (N = 1066). The characteristics of the selected subjects are summarised in Table 1. Statistical significance testing was performed to compare the chosen subjects (N = 1066) with the APRHI cohort (*N*_*a*_ = 4535) using a two-sample, two-sided t-test for continuous variables, and the Wilcoxon signed-rank test for variables ‘smoking’ and ‘treated for hypertension’. The difference was not statistically significant (p>0.05) for all gender-stratified variables in Table 1 except male smokers (p = 0.0095).

**Table 1:** Population characteristics from the chosen subset of APHRI (N = 1066). Statistical significance testing was performed to investigate this chosen subset from the full APHRI cohort (*N*_*a*_ = 4535). Only male smokers were significantly different (p = 0.0095; Wilcoxon signed-rank test). BP indicates blood pressure.

Feature	Male	Female
N	48.8% (520)	51.2% (546)
Age, mean±sd, (years)	50.6±14.1	48.2±13.4
Current Smoker,%(n)	40.6% (211)	5.1% (28)
Systolic BP, mean±sd, (mmHg)	126.1±20.1	122.4±20.5
Diastolic BP, mean±sd, (mmHg)	77.2±11.5	76.2±10.7
Glucose, mean±sd, (mg/dl)	99.8±28.7	102.4±35.9
Total Cholesterol, mean±sd, (mg/dl)	177.8±40.4	191.1±38.7
Treated for hypertension,%(n)	13.8% (72)	14.8% (81)

According to the 2009 NPCDCS guidelines, a patient with less than a 10% 10-year risk score should be considered to be at low risk of developing CVD. If the risk score is between 10% to <20%, the patient should be considered to have a moderate risk of developing CVD. Patients with risk scores between 20% to <30% should be considered to be at high risk while those above 30% should be deemed to be at ‘very high risk’ of developing CVD [5]. Treatment for high risk subjects was initiated either if their CVD risk score was above 30%, or if the CVD risk score for an individual was between 20% up to 30% and their systolic blood pressure was over 140 mmHg [9]. The group of subjects who are at high risk and require treatment is henceforth referred to as *T*_*HR*_. For objective 1, we use the NPCDCS guidelines for the interpretation of a risk range and highlight *T*_*HR*_ subjects who are clinically more relevant. For objective 2, we develop POC algorithm using patients belonging to all categories of risk.

### Identifying patients who are likely to benefit from a cholesterol test

### Feature Selection

A total of 40 features that have been reported in CVD literature were chosen from the APRHI dataset for input into a feature selection algorithm, the Maximum Relevance Minimum Redundancy (mRMR) approach [10]. The mRMR criterion penalises the pairwise mutual information between the features while maximizing the mutual information between features and class labels. It is defined as

$$\begin{array}{c} mRMR=\max_{S}\{\frac{1}{\left|S\right|}\sum_{f_{i}\in S}I\left(f_{i};c\right)-\frac{1}{{\left|S\right|}^{2}}\sum_{f_{i},f_{j}\in S}I\left(f_{i};f_{j}\right)\} \end{array}$$

where *S* is the feature set, *I* is the mutual information between feature *f*_*i*_ and class *c* or between features *f*_*i*_ and *f*_*j*_.

From the top 20 features ranked using this criterion, we selected those features which are clinically relevant features that were either easy to measure, low-cost, or easy to access. The feature set included the following 10 variables: gender, age, current smoker, past history of diabetes, past history of high cholesterol, Body Mass Index (BMI), Systolic Blood Pressure (SBP), Diastolic Blood Pressure (DBP), treatment for hypertension and blood glucose.

### Development of model for identifying TC candidates

Data from a total of 1066 patients were divided into training and testing sets in a ratio of 70:30. The WHO HI and LI risk prediction charts were implemented in the Matlab programming environment (code available as an additional file to this paper). The predicted CVD risk ranges were then obtained from both the HI and LI risk prediction charts on the data. The subpopulation benefitting from a TC test comprises patients for whom the predictions by LI and HI risk prediction charts differed, and this was the target outcome of our model. Three different classification approaches were trained and tested; Support Vector Machine (SVM), Random Forest (RF), and Logistic Regression (LR). SVMs were originally developed by Vapnik [11] and perform classification by constructing a hyperplane in a high dimensional space that maximally separates members of one class from another in the training set. A kernel function helps to find the separating hyperplane in the feature space without the need to explicitly represent the space. SVMs have shown excellent performance in different applications [12]. For our task of identifying the subpopulation, we used a linear kernel SVM, defined by *k*(*x*_*i*_, *x*_*j*_) = (*x*_*i*_.*x*_*j*_+1)^*d*^. The SVM’s capacity *C*, a parameter allowing the trade-off between model complexity and classification error, needs to be optimised and this was done using a grid search of the feature space in the training set. An internal four-fold cross validation was used to find the best *C* by optimising the area under the curve (AUC). The libSVM implementation by Chang and Lin [13] was used in the Matlab programming environment. A Random Forest is an ensemble-technique that uses bagging (bootstrap aggregation) and random feature selection to perform classification [14]. The RF technique is state-of-the-art and offers advantages such as robustness to outliers and missing data, fewer tuning parameters, automatic determination of feature importance, and high classification accuracy [15]. We used the Matlab implementation of the randomforest R package [16]. Logistic regression is a standard baseline approach with a probabilistic framework for classifying binary outcomes. The regularised logistic regression was trained and tested using an *L*_1_ penalty [17]. This removes redundant features thereby providing a method of feature selection and helps build a *parsimonious* model.

### Model Validation

Four-fold cross validation was performed on the training set to optimise hyperparameters and obtain the best performing model. For RFs, the Out of Bag (OOB) samples in the training set were used to optimise the number of trees and number of variables sampled for each split. The OOB samples are those samples that are unused during the construction of each tree. They can therefore act as an internal test set for each tree and the resulting error estimate has been proven to be unbiased [18]. For regularised LR, the mean of the regularisation parameter was derived through the four-fold cross validation. This was used to re-train the classifier and performance on the training set was estimated. The final model performance for all classifiers was evaluated on the test set.

### Evaluation Metrics

To evaluate the model, we use the area under curve (AUC), which is a measure of the classifier’s discriminative ability. We also used the F-score to pick thresholds on the Receiver Operating Characteristics (ROC) curve. The F-score is the harmonic mean of the precision and recall, as defined below.

#### Recall

The recall or sensitivity (Se) is the true positive rate, or the number of times a patient belonging to the subpopulation is correctly identified. This means the number of times a patient for whom a TC test is beneficial for CVD risk prediction by the WHO/ISH models is correctly identified. It is defined as follows:

$$\begin{array}{c} Recall=\frac{TP}{TP+TN} \end{array}$$

where TP indicates true positives and TN indicates true negatives.

#### Specificity

The specificity (Sp) is the true negative rate, or the number of times a patient who does not belong to the subpopulation is correctly identified.

$$\begin{array}{c} Specificity=\frac{TN}{TP+TN} \end{array}$$

where TN indicates true negatives and TP indicates true positives.

#### Precision

The precision or positive predictive value is the proportion of true positives amongst all positive results and is defined as

$$\begin{array}{c} Precision=\frac{TP}{TP+FP} \end{array}$$

where FP indicates the number of false positives.

#### F-score

The generalised F-measure is given by

$$\begin{array}{c} F_{\beta }=\left(1+\beta^{2}\right).\frac{precision.recall}{\beta^{2}.precision+recall} \end{array}$$

where *β* measures the effectiveness of retrieval with respect to someone who attaches *β* times as much importance to recall as precision. We report the F_1_, F_2_, and F_3_ scores when *β* = 1, 2, and 3 respectively.

Ethics: The APRHI project was approved by the Institutional Ethics Committee of the CARE Hospital, Hyderabad in India and the University of Sydney Human Research Ethics Committee, NSW, Sydney. Participants provided informed, written content to contribute data to the study. The analysis carried out in this article used de-identified, anonymised patient data from the APHRI project.

## Results

### Objective 1: Relative performance of LI and HI WHO/ISH CVD risk prediction charts

**Fig 1:**
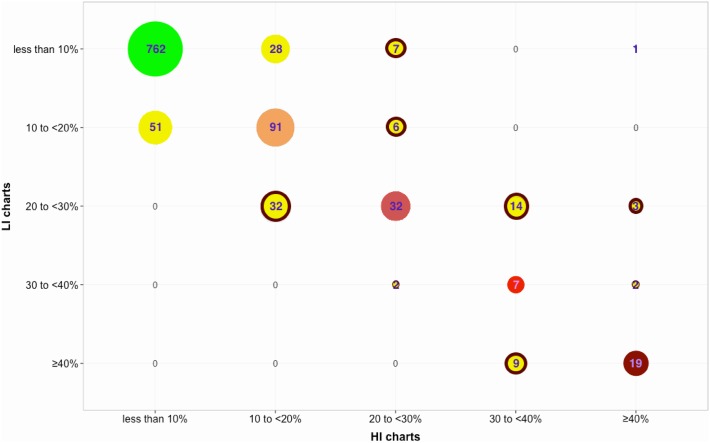
CVD risk prediction using the LI and HI WHO/ISH risk prediction charts on the chosen subset of APHRI data (N = 1066). The five CVD risk ranges for whom the predicted risk using LI and HI WHO/ISH risk prediction charts concurred, are colour coded as follows: less than 10% risk in green; 10 to <20% risk in orange; 20 to <30% risk in red; 30 to <40% in light red; and ≥ 40% risk in maroon. Subjects for whom the predictions differed are highlighted in yellow circles with those that can be *T*_*HR*_ having a red highlight.

Out of 1066 subjects, the LI and HI risk prediction charts misclassified 155 subjects (or 14.5%) relative to each other as shown in Fig 1. Statistical significance testing was performed using the non-parametric Friedman’s test, which is suitable for ordinal data (since the WHO/ISH charts predict in quantised ranges). The choice of WHO/ISH risk prediction chart was statistically significant (p = 0.008;*χ* ^2^ = 7.03).

The predictions from LI model resulted in 108 subjects being classified as *T*_*HR*_ while the HI model classified 88 subjects to be *T*_*HR*_ upon application of the previously described NPCDCS clinical guidelines. Both LI and HI models concurred on 80 subjects to be *T*_*HR*_ (p = 0.2568 using Friedman’s test, implying the choice of prediction model is not significant). However, they disagreed on 36 subjects overall (p<0.01 using Friedman’s test, implying choice of prediction model is significant), which is 31% of all subjects identified as *T*_*HR*_ by either HI or LI model. If it can be assumed that the HI model is more accurate on account of additional information required for prediction, we observe 28 *T*_*HR*_ subjects are misclassified. This implies that the LI model over-predicts high risk subjects requiring treatment as compared to the HI model.

Table 2 shows the statistical characteristics of the misclassified subjects. Statistical significance testing was performed to compare the subpopulation (n = 155) with the larger dataset of chosen subjects (N = 1066) using a two-sample, two-sided t-test for continuous variables, and the Wilcoxon signed-rank test for variables ‘smoking’ and ‘treated for hypertension’. The difference was statistically significant (p<0.05) for all gender-stratified variables in Table 2 except male total cholesterol measurements (p = 0.0530), female diastolic blood pressure levels (p = 0.0672), female blood glucose measurements (p = 0.1355), and female smokers (p = 0.7484). We can observe that the mean risk factors levels for SBP are at the thresholds of what is deemed normal and abnormal as per clinical guidelines. This indicates that those subjects who will be the subject of step thresholds (as opposed to a smooth change) are prone to misclassification.

**Table 2:** Statistical characteristics of the subpopulation (n = 155) who are likely to benefit from TC testing. To investigate variables that were different between the subpopulation and chosen subjects (N = 1066), significance testing was performed. The difference was statistically significant (p<0.05) for all gender-stratified variables except male total cholesterol measurements (p = 0.0530; t-test), female diastolic blood pressure levels (p = 0.0672; t-test), female blood glucose measurements (p = 0.1355; t-test), and female smokers (p = 0.7484; Wilcoxon signed-rank test).

Feature	Male	Female
n	57.4% (89)	42.6% (66)
Age, mean±sd, (years)	62.1±9.4	62.5±9.1
Current Smoker,%(n)	53.9% (48)	6.1% (4)
Systolic BP, mean±sd, (mmHg)	140.7±23.4	140.8±21.6
Diastolic BP, mean±sd, (mmHg)	81.7±13.2	78.8±12.3
Glucose, mean±sd, (mg/dl)	108.2±41.4	109.9±52.2
Total Cholesterol, mean±sd, (mg/dl)	187.4±57.5	206.1±61.0
Treated for hypertension,%(n)	29.2% (26)	34.8% (23)

### Objective 2: Models to identify subjects who benefit from TC testing

**Fig 2:**
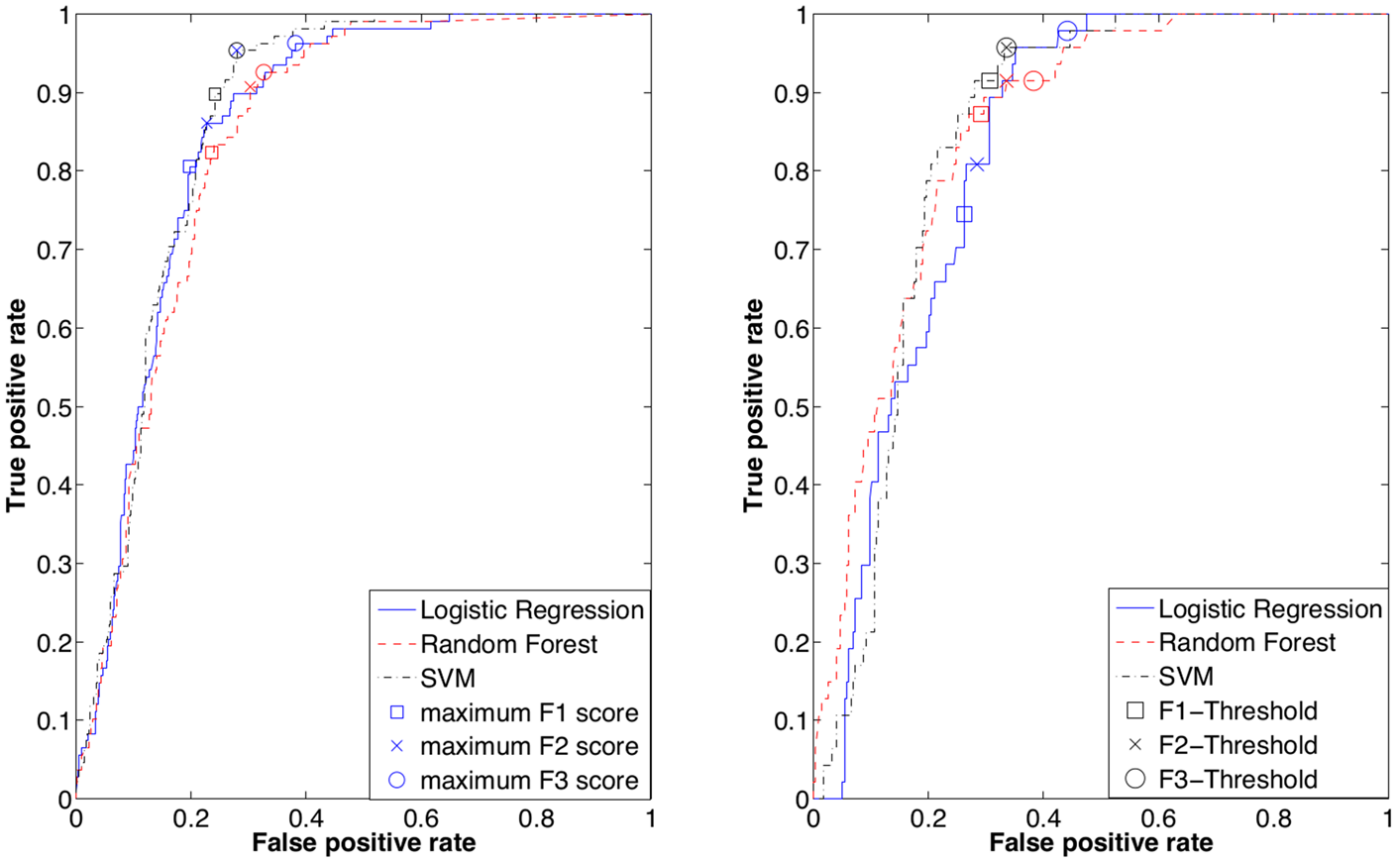
Receiver Operating Characteristic Curves for the SVM, RF, and RLR models. The F1, F2, and F3 scores were computed for every point on the ROC curve from the training dataset and Plot **(a)** illustrates thresholds where the scores were maximum. The Out of Bag samples during training were used to obtain the ROC curve for RF since the performance on the training data was perfect (AUC 1.00). Plot **(b)** represents ROC of the test data where thresholds chosen from the training data are marked.

The out of sample performance of the classifiers is summarised in Table 3. RF has the highest AUC of 0.85 while the performance of SVMs is comparable (AUC 0.84). The Receiver Operating Characteristics (ROC) for the training and test data are shown in Fig 2. The F_1_, F_2_, and F_3_ scores were computed and Fig 2a shows thresholds where the scores were maximal on the training data. Subsequently, the same thresholds were applied on the ROC curve of the test data (in Fig 2b) and the resulting sensitivity and specificity are described in Table 3. For RF, the OOB samples during training were used to calculate the F-scores since the AUC on the training data was perfect (AUC 1.00). The F_1_ scores offer a balance between sensitivity and specificity, while F_3_ scores emphasise sensitivity over specificity. For example, performance of SVMs on test data shows that at the maximum F_1_ score, a sensitivity of 91% and specificity of 69% was achieved while at the maximum F_3_ score, we obtain 96% sensitivity and 66% specificity.

**Table 3:** Discriminative ability of Support Vector Machine (SVM), Random Forest (RF), and L1-Regularised Logistic Regression (RLR) to classify patients likely to benefit from a TC test. The F_1_, F_2_, and F_3_ scores were obtained from the training data and used to threshold the out of sample test data (indicated by †).

**Classifier**	**AUC**	**F_1_ measure**			**F_2_ measure**			**F_3_ measure**		
		F_1_	Se	Sp	F_2_	Se	Sp	F_3_	Se	Sp
		score	(%)	(%)	Score	(%)	(%)	Score	(%)	(%)
*SVM*	0.87	0.54	90	76	0.72	95	72	0.82	95	72
*SVM†*	0.84		91	69		96	66		96	66
*RF*	0.84	0.51	82	76	0.68	91	70	0.78	93	67
*RF†*	0.85		87	71		91	66		91	62
*RLR*	0.86	0.54	81	80	0.69	86	77	0.79	96	62
*RLR†*	0.82		75	74		81	71		98	56

**Fig 3:**
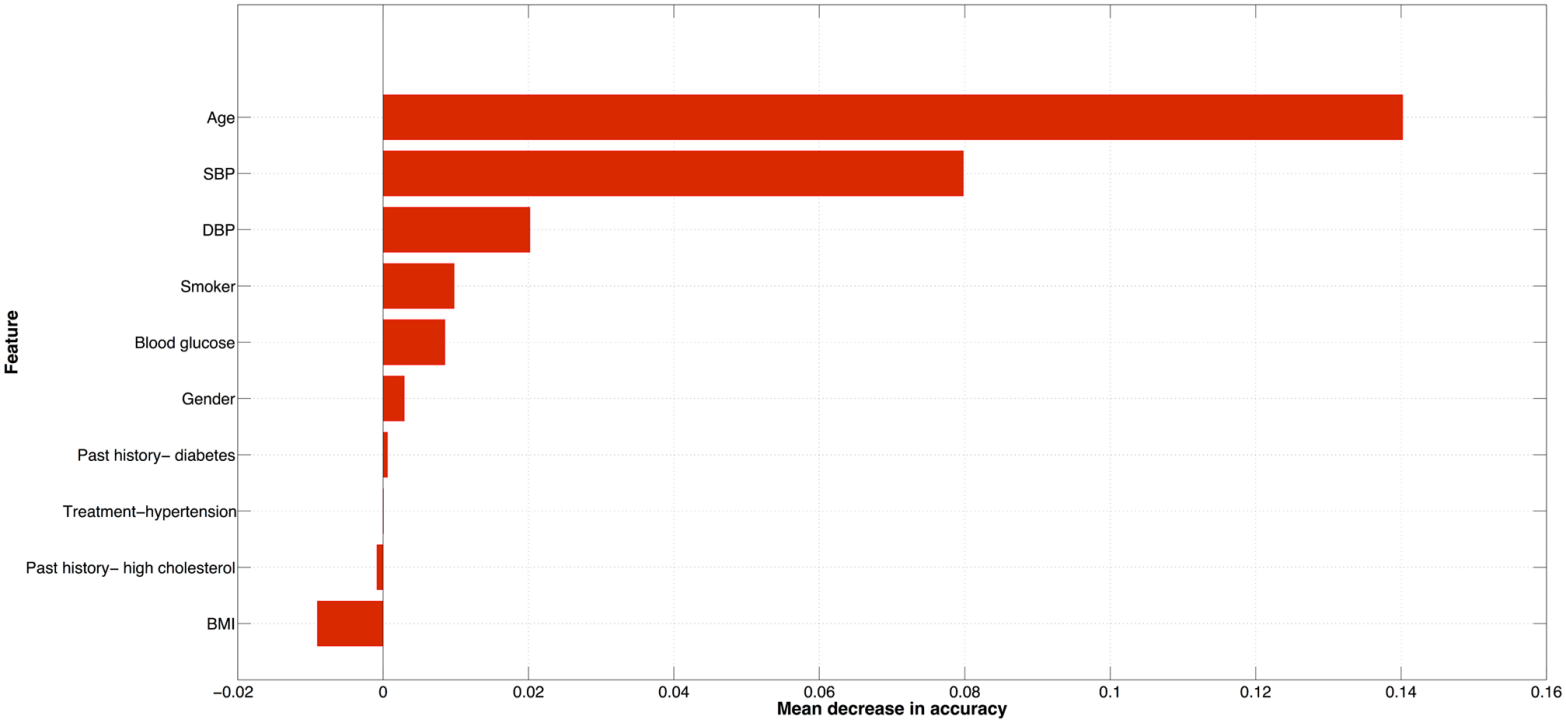
Variable importance ranked according to the mean decrease in accuracy using the RF OOB samples.

The regularised logistic regression model picked two features as non-redundant from the entire feature set, namely age and SBP. The coefficients of the RLR model are given in Eq 1. A similar inference on the most predictive features can be drawn based on the variable importance plot for the RF as shown in Fig 3. The importance plot shows the mean decrease in accuracy caused by a feature using the OOB samples. The larger the mean decrease in accuracy, the more important the feature is deemed to be—and according to Fig 3, age was most important followed by SBP.

$$\begin{array}{c} \begin{array}{c} logit(c)=-5.6554+0.0416*Age+0.0132*SBP \end{array} \end{array}$$ (1)

where *c* is the probability for a patient to require a cholesterol test. *c* can be thresholded using the F1 or F3 score depending on the emphasis between recall and precision one wishes to have. The supporting file to this article illustrates the use of this POC approach through worked examples (see S1 File).

## Discussion

We have shown that in a rural Andhra Pradesh population, the version of WHO/ISH algorithm is important because there is a 14.5% difference in the predicted risk ranges between the LI and HI CVD risk charts. We also observed that the mean values of risk factors (especially SBP) in Table 2 are higher as compared to the chosen APHRI population (Table 1), and close to the cut-off for what is deemed normal and abnormal as per clinical guidelines. This indicates that it is important to accurately stratify the risk in order to avoid missing out or under-treating people who may be at a higher risk and vice versa. The statistical approaches presented here can offer population-specific insights to resource allocation and cost saving. In countries like India, the cost of a cholesterol test has been reported to vary between $4 to $30 [19]. Although the Indian government’s health spending has been increasing, the per capita expenditure is still only $61 per annum [20]. This implies that cholesterol testing has to be selective when population-wide strategies such as CVD risk screening is performed. Moreover, access to POC cholesterol testing is difficult and it is often the most expensive part of CVD risk assessment. Also, there can be substantial treatment implications for patients who are misclassified. If the HI model is assumed to be more accurate, screening using the LI chart will over predict risk for people at high CVD risk. We observed 28 subjects (which is 32% of those at *T*_*HR*_ according to HI model) to be misclassified as *T*_*HR*_. This can have two major adverse implications in terms of over-treatment and prioritising patients for re-assessment/follow-up. We estimate the average cost of treating a high risk patient is Indian Rupees (INR) 15 to 25 per day (or INR 450 to 650 per month). Out of 1.252 billion people in India [21], 368 million (29.4%) are aged over 40 years [22]. A minimum of 15% of those above 40 years or 55 million people are at high CVD risk (unpublished data). Therefore we have 18 million people who can be over-treated if the LI risk prediction charts are used, which would result in an expenditure of INR 8 to 13 billion a month. With a projected 61.5 million cases in India [23], with over 3 million new cases each year, our approach could potentially save substantial money in testing and tens of thousands of hours of human labour each year.

**Fig 4:**
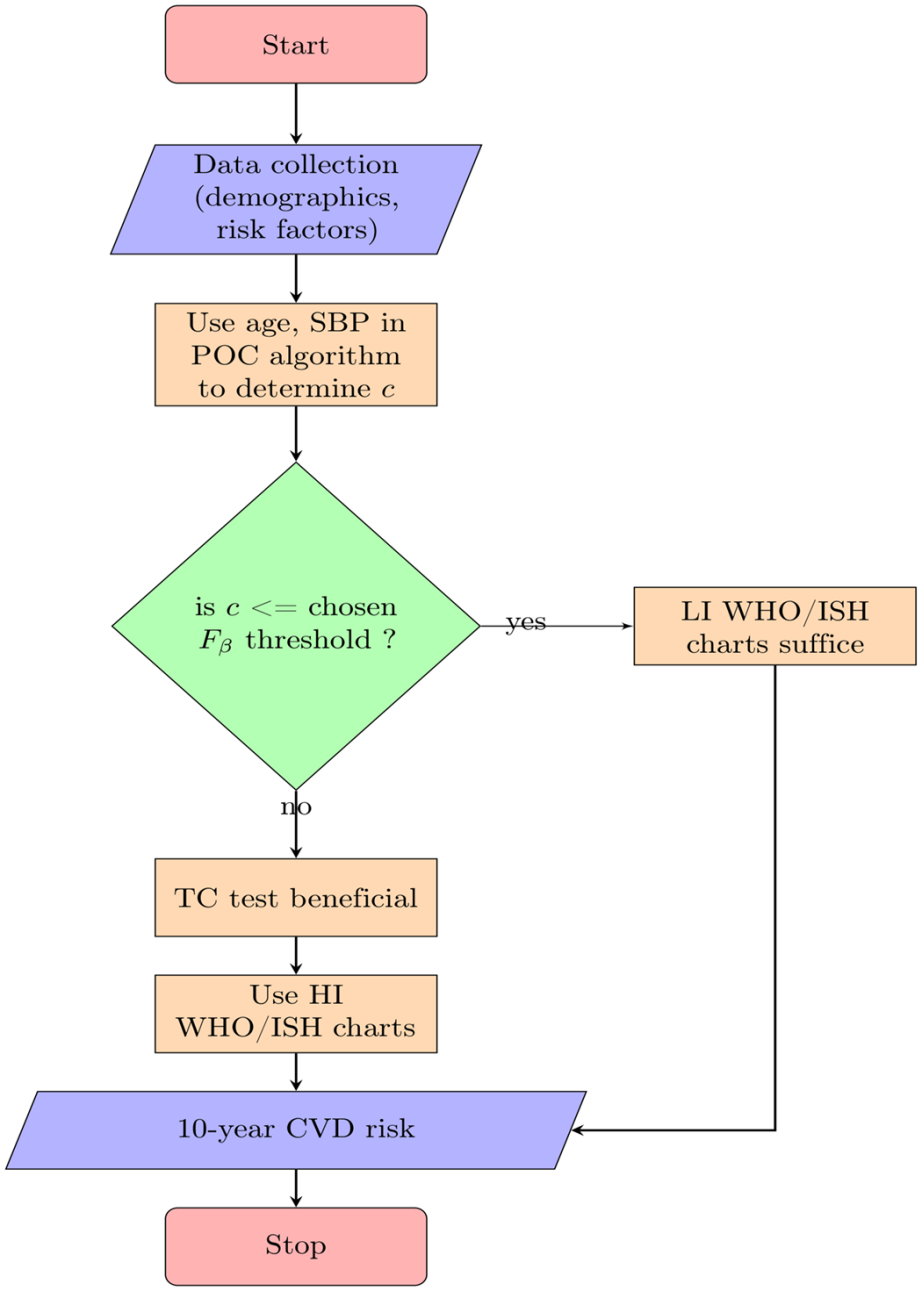
Flowchart illustrating the process of decision support using the POC algorithm when performing risk assessment using the WHO/ISH risk prediction charts.

The advent of electronic tools such as tablets is an opportunity to package advanced statistical machine learning algorithms to assist risk predictions at point-of-care. One such example is the SMARThealth India study, which investigates the effectiveness of clinical decision support for minimally trained health workers through mobile devices for CVD risk prediction and management [24]. Such a mobile based decision support system could be programmed to identify if the patient would benefit from a cholesterol test before CVD risk is computed. For instance, at point-of-care, a health worker can perform data collection on the patients age (and other demographics) and blood pressure. Our algorithm, which when trained and installed on the mobile device, can then use the age and SBP alone to perform decision support on whether a total cholesterol test is necessary prior to CVD risk prediction with the WHO/ISH risk prediction charts. This process for decision support can be understood further through the flow-chart illustrated in Fig 4

One of the main limitations of the work presented here is the fact that the dataset was acquired from a southern India population which might not be representative of the whole of India because of the large genetic diversity [25] and socio-economic inequities. However, rural regions of India are possibly in different stages of epidemiological transition. For instance, BP levels in Andhra Pradesh are largely identical to those reported in urban India [26][27] for similar age groups, which suggests that the Andhra Pradesh region is at an advanced stage of transition. Chow et al. (2008) concur in this view by suggesting that rural regions in India are likely to develop risk factors levels comparable to results from APHRI [8]. The lack of recorded CVD outcomes in the APHRI study is a barrier to validate both LI and HI models of WHO/ISH risk prediction charts with a gold standard. However, it is worth noting that there have been little or no large-scale prospective studies with recorded CVD events in India, especially ones that also record lipid profiles [8]. Also, a large number of patients in the dataset were of low CVD risk according to the WHO/ISH risk prediction charts. The WHO/ISH CVD risk prediction charts do not discuss if the LI model or HI model is more accurate in predicting CVD risk. We therefore make the assumption that the HI model is more accurate since it requires more information for prediction.

## Conclusion

We have demonstrated that the choice of LI or HI WHO/ISH models (for SEAR-D) significantly affects CVD risk prediction on rural Indian residents with a difference of 14.5% (n = 155; p = 0.008) overall. In terms of clinical relevance, LI and HI models disagree on *T*_*HR*_ status of 36 patients (31% of all subjects identified as *T*_*HR*_ by either model). If the HI model is assumed to be more accurate, the LI model is observed to overpredict CVD risk for *T*_*HR*_ patients. Our POC test leverages two patient-specific risk factors, namely age and SBP, that are collected anyway during risk assessment with the WHO/ISH charts. This can assess the benefit of total cholesterol testing before risk computation and subsequently pre-determine whether the LI model is preferential to the HI model. Our analysis resulted in out-of-sample AUCs of 0.85 (RF), 0.84 (SVM), and 0.82 (RLR) with upto 98% sensitivity. An understanding of the differences in risk prediction between the LI and HI models, and adoption of a pre-screening POC test to assess the benefit of a TC test, can aid planning for resource-allocation and saving costs for large-scale screening programmes.

## Supporting Information

S1 File Worked examples for POC algorithm. We present two worked examples of using the simplified POC test in routine risk assessment for determining if a TC test will benefit CVD risk prediction using the WHO/ISH charts. Patient data is taken from our testing set of the APHRI dataset.
**Fig A**—A 3D plot illustrating the variation of age and systolic blood pressure with predicted probabilities from RLR model.
**Fig B**—Low information WHO/ISH CVD risk charts.
**Fig C**—High information WHO/ISH CVD risk charts.
(PDF)

S2 File Code for WHO/ISH charts. A MATLAB implementation of the 10-year CVD risk scores using the WHO/ISH CVD risk prediction charts for SEAR-D.
(ZIP)
