# Supplementary material for: Implications of Cardiovascular Disease Risk Assessment Using the WHO/ISH Risk Prediction Charts in Rural India
Source: PLoS One. 2015 Aug 19;10(8):e0133618. doi: 10.1371/journal.pone.0133618 (PMC4545825; doi:10.1371/journal.pone.0133618)
Supplement: S1 File — We present two worked examples of using the simplified POC test in routine risk assessment for determining if a TC test will benefit CVD risk prediction using the WHO/ISH charts. Patient data is taken from our testing set of the APHRI dataset. Fig A—A 3D plot illustrating the variation of age and systolic blood pressure with predicted probabilities from RLR model. Fig B—Low information WHO/ISH CVD risk charts. Fig C—High information WHO/ISH CVD risk charts. (PDF) [file pone.0133618.s001.pdf]

Evaluating cardiovascular disease risk using the WHO/ISH risk prediction charts for South East Asian Regions D

Arvind Raghu, Devarsetty Praveen, David Peiris, Lionel Tarassenko, and Gari Clifford

Supporting Information File 1

We present two worked examples of using the POC simplified test for determining if a TC test will benefit CVD risk prediction using the WHO/ISH charts. Patient data is taken from our testing set of the APHRI dataset.

Example 1

Consider a 70 year old male patient whose data is being collected at point of care. He is a non-smoker and weighs 49kg and is 162.5 cm tall. The patient has two consecutive blood pressure measurements taken which are recorded to be 137/87 mmHg and 138/84 mmHg. He also records a random blood glucose level of 79 mg/dl.

1. Prior to determining 10-year CVD risk, we can use equation (1) as described in the paper,

   $$logit(c) = -5.6554 + 0.0416 * Age + 0.0132 * SBP \quad (1)$$

   where c is the probability for a patient to require a cholesterol test.

2. The patient's mean SBP is calculated to be 137.5 mmHg.

   $$\begin{aligned} logit(c_1) &= -5.6554 + 0.0416 * (70) + 0.0132 * (137.5) \\ c_1 &= \frac{1}{(1 + e^{-0.9284})} \\ c_1 &= 0.2832 \end{aligned}$$

3. The threshold corresponding to the maximum F3 score, which has a high sensitivity, is 0.1215. Alternatively, the threshold corresponding to the F1 measure may also be considered (0.1762) if the user desires a balance between sensitivity and specificity. Both thresholds are below c_1 indicating

that a TC test will be beneficial for risk estimation which implies that it is preferable to use the WHO/ISH HI charts.

The patient's complete medical data reveals a TC measurement to be 221 mg/dl. Hence the 10-year risk computed using the WHO/ISH LI charts would have been 20 to <30% risk while risk computed using the HI charts would be 30 to <40% risk. As a side note, if the patient had been assessed for risk only using the WHO/ISH LI charts, the NPCDCS guidelines would not have classified the patient as requiring treatment (10-year CVD risk would have been between 20 to <30% and SBP<140mmHg). On the other hand, the patient's risk according to the HI charts clearly mandates that he ought to be high risk and requiring treatment.

Example 2

Consider a 48-year old male patient, non-smoker with height 158cm and weight 61kg. He has a mean BP of 154/89 mmHg from two consecutive readings and a glucose level (random) of 110 mg/dl.

1. Using equation (1), we find

$$\begin{aligned} logit(c_2) &= -5.6554 + 0.0416 * (48) + 0.0132 * (154) \\ c_2 &= \frac{1}{(1 + e^{-1.6258})} \\ c_2 &= 0.1644 \end{aligned}$$

2. If we choose the threshold corresponding to the maximum F3 score (0.1215), a TC test and risk estimation by the WHO/ISH HI charts are recommend. However, the threshold for maximum F1 score (0.1762) which offers higher specificity would say the TC test won't be necessary and WHO/ISH LI charts would suffice.

The patient's complete medical data tells us his recorded TC was 208 mg/dl. Both the WHO/ISH LI charts and HI charts would estimate the 10-year risk to be less than 10% risk.

The variation of age, SBP, and c is shown through Fig. A.

WHO/ISH risk prediction charts

The LI and HI WHO/ISH risk prediction charts for SEAR D are illustrated through Fig. B and Fig. C respectively.

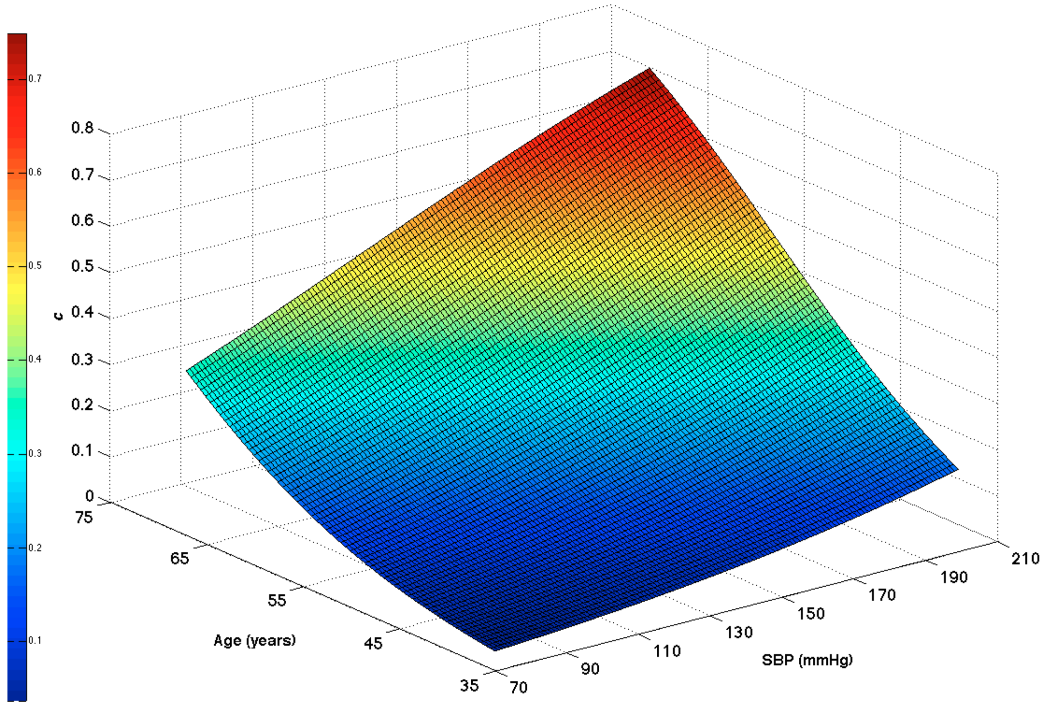

Figure A: The variation of c with age and systolic blood pressure.

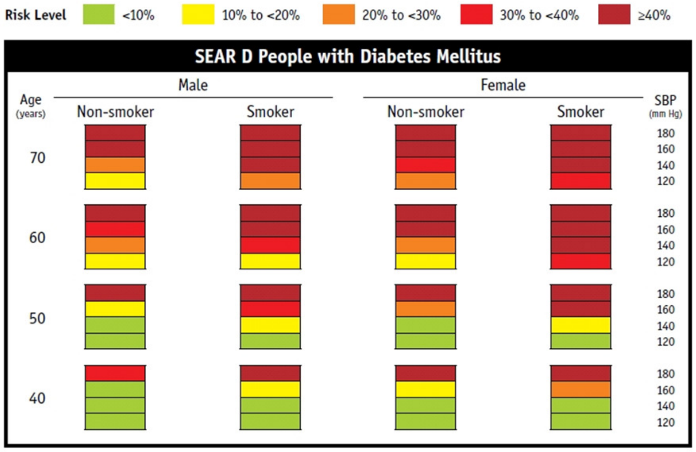

(a)

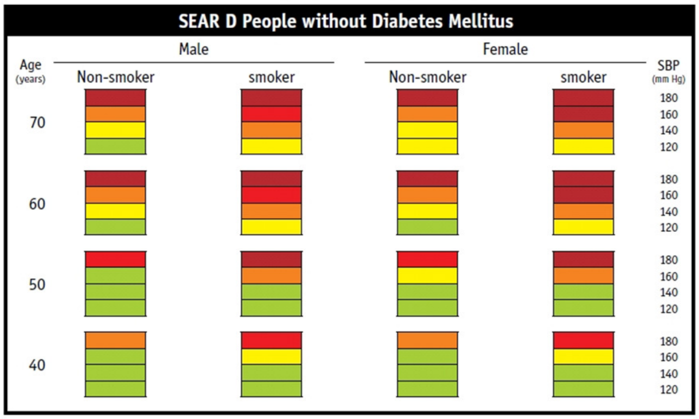

(b)

Figure B: Low information version of WHO/ISH CVD risk chart for SEAR D.

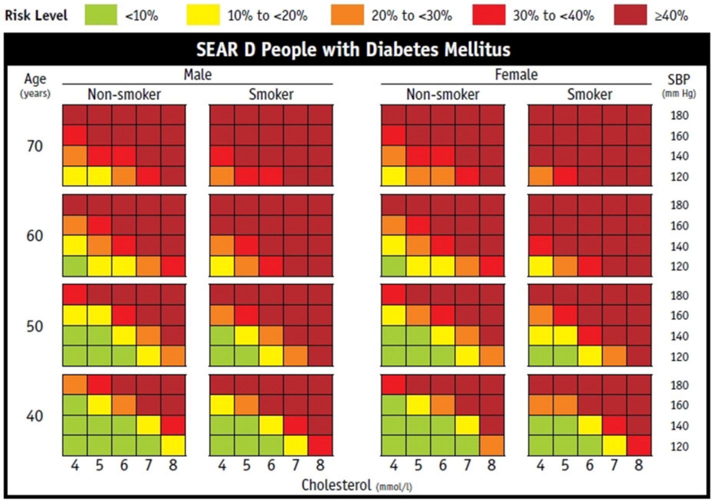

(a)

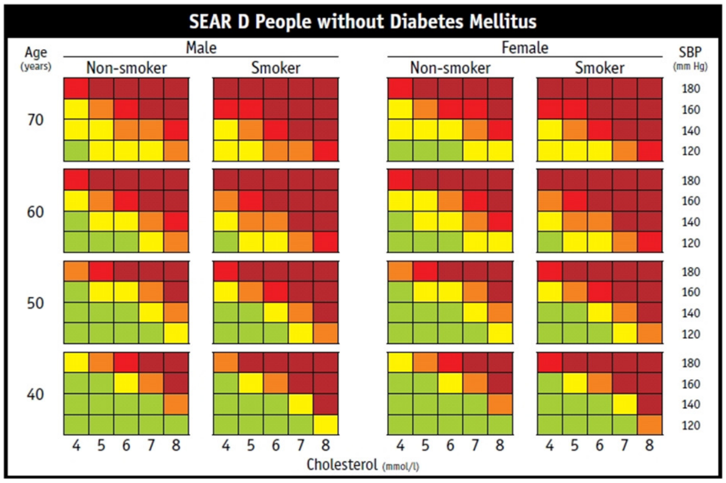

(b)

Figure C: High information version of WHO/ISH CVD risk chart for SEAR D.
