# Supplementary material for: Implications of Cardiovascular Disease Risk Assessment Using the WHO/ISH Risk Prediction Charts in Rural India
Source: PLoS One. 2015 Aug 19;10(8):e0133618. doi: 10.1371/journal.pone.0133618 (PMC4545825; doi:10.1371/journal.pone.0133618)
Supplement: S2 File — A MATLAB implementation of the 10-year CVD risk scores using the WHO/ISH CVD risk prediction charts for SEAR-D. (ZIP) [file pone.0133618.s002.zip › Code/NPCDCS_guidelines_India.pdf]

National Programme for Prevention and Control of Diabetes, Cardiovascular Disease and Stroke

A Manual for Medical Officer

Developed under the Government of India – WHO
Collaborative Programme 2008-2009

Contents: **Page No.**

1. Introduction 3
2. Role of medical officer to promote healthy lifestyle in community 5
3. Risk factors of NCDs and their relationship 6
4. Cardiovascular disease risk assessment and management 8
5. Treatment guidelines for
   - 5.1. Diabetes 17
   - 5.2. Hypertension 28
   - 5.3. Hypercholesterolemia 33
   - 5.4. CAD 36
   - 5.5. Stroke 47
6. Suggested Reading 51
7. List of Experts and Institutions involved 52

Section 1: INTRODUCTION

Non-communicable diseases (NCD), also known as chronic diseases include cardiovascular diseases, diabetes, stroke, most forms of cancers and injuries. Such diseases mainly result from lifestyle related factors such as unhealthy diet, lack of physical activity and tobacco use. Changes in lifestyles, behavioural patterns, demographic profile (aging population), socio-cultural and technological advancements are leading to sharp increases in the prevalence of NCD. These diseases by and large can be prevented by making simple changes in the way people live their lives or simply by changing our lifestyle.

Magnitude of NCD burden in India

During the year 2005, NCD accounted for 53% of all the deaths in the age group 30-59 years in India. Of these, 29% were due to cardiovascular diseases; It is estimated that, by 2020, cardiovascular disease will be the largest cause of disability and death, as a proportion of all deaths in India. In 2003 alone, in India, there were approximately 30 million people suffering from coronary heart disease.

Diabetes which is a major risk factor for chronic disease on it own causes increased death and disability. According to the Diabetes Atlas 2006 published by the International Diabetes Federation, the number of people with diabetes in India is currently around 40.9 million and is expected to rise to 69.9 million by 2025, unless urgent preventive steps are taken. Similarly, 118 million people were estimated to have high blood pressure in the year 2000 which is expected to go up to 213 million in 2025. Not only this, **Indians succumb to diabetes, high blood pressure and heart attacks 5-10 years earlier than their western counterparts, during their most productive years. This leads to considerable loss of productive years, to the country.** It has been estimated that, by the year 2030, India will lose approximately 17.9 million potentially productive years which is higher than the expected combined loss in China, Russia, USA, Portugal and Brazil. This translates into a huge economic loss as high as 237 billion dollars by the year 2015. Development of diabetes and heart attacks at an early age is not largely because of environmental causes such as low consumption of fresh fruits and vegetables along with other unhealthy diet, increasing use of tobacco, and higher prevalence of sedentary life-style.

To contain the increasing burden of Non-Communicable Diseases, Ministry of Health and Family welfare, Government of India, has launched the National Programme on Prevention and Control of Diabetes, Cardiovascular diseases and Stroke (NPDCS) with the following objectives:

Objectives of NPDCS:

- Prevention and control of NCDs
- Awareness generation on lifestyle changes
- Early Detection of NCDs
- Capacity Building of health systems to tackle NCDs

Pilot phase has been launched in 10 states in 10 districts (January 2008) focused on health promotion and health education advocacy at various settings.

The following Interventions are planned in the programme:

i) Health promotion and health education for community,

ii) Early Detection of Persons with High Levels of Risk Factors (at the risk of developing disease) through screening

iii) Strengthening health systems at all levels to tackle NCDs and improvement of quality of care including treatment of Sleep disorders and augmenting facilities of dialysis.

The following components are envisaged in the programme

1. District NPDCS Programme (626 Districts)
2. NCD Focal Centres at Medical College (54 Medical Colleges)
3. State/UT NCD Cell (35)
4. National NCD Cell at Center
5. IEC/ BCC
6. Capacity Building and Research
7. Inter-sectoral Convergence
8. Monitoring (including MIS) and Evaluation

The programme shall be implemented in 626 districts in all states/ UTs in India with the interventions at Medical Colleges (54), Districts hospitals, CHCs (3035), PHCs (16778) and all Sub centres through community level activities. The programme shall be leveraging the strengths of NRHM at the primary and secondary health care set up (SC/PHC/CHC/District Hospitals) through convergence, Need based training, Private Public Partnership and NGO interventions in school, workplace and community settings. USHA or any other available Health worker as well as NGO's and Private Practitioners shall be roped in for providing effective promotion, prevention and control strategies on NCD's and its risk factors for urban areas.

Section 2: ROLE OF MEDICAL OFFICER IN NPDCS

1. Health promotion activities - educate regarding common risk factors, promote health in different settings (healthy workplace, health promoting schools) to prevent the emergence and reduce the existing risk factors in the community

2. Risk assessment and management through opportunistic screening

3. Motivate and create role models in the community

4. Work closely with other sectors/ departments for NCD prevention

5. Management of patients suffering from Diabetes, CVDs and Stroke referred from different centers

6. Establish an effective referral mechanism (two way) with the nearest medical colleges

7. Supervision of the activities undertaken by paramedical workers

8. Assist resource centers/ institution in organizing the training for different cadre of health workers

How to develop Health promoting Schools?

Schools play a vital role in the overall development of a child into a competent adult who contributes usefully to the society. Health is an important aspect of development of children and education is an important determinant of health. Almost all children attend school at sometime during their lives and spend 6-7 hours of their time everyday in that learning environment. Apart from this, the school curriculum can have a substantial influence on health promoting behaviours. Schools have profound influence on thinking patterns and behaviors of children, their families, and the interventions can reach generations of children.

A health promoting school (HPS) is a school community that takes action and gives priority to create an environment that will have the best possible impact on the health of students, teachers and other school staff. Such a programme can be successful only if it is owned and initiated by educational sector in close association with health and other related sectors like sports, youth affairs, mass media etc.

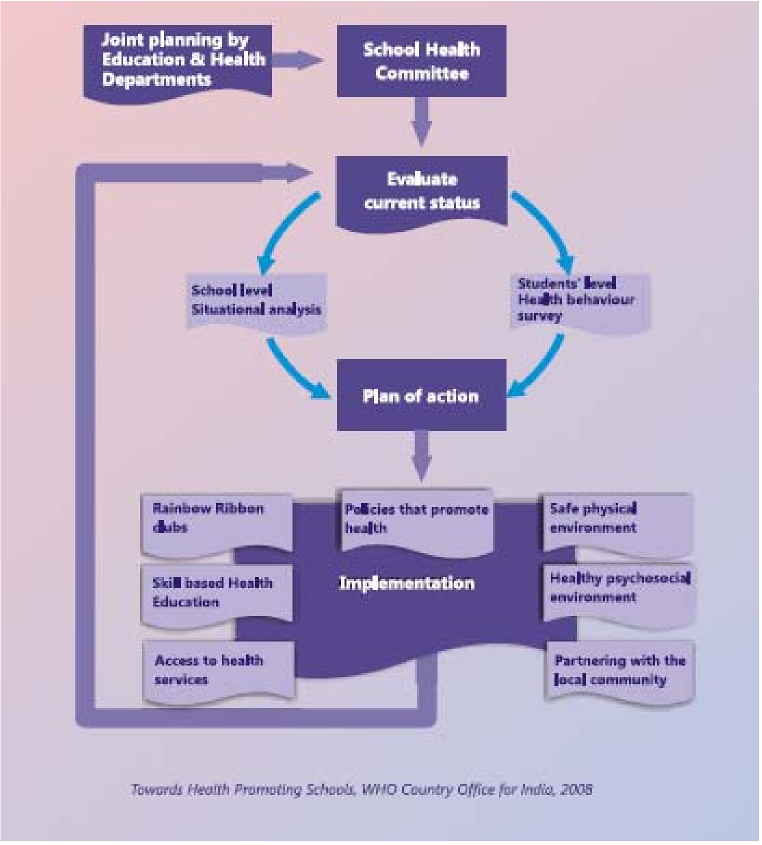

Towards Health Promoting Schools, WHO Country Office for India, 2008

As medical officer you should play a proactive role to initiate implementing the concept of HPS in your area of work with the help of paramedical workers, teachers and community volunteers.

These are the following steps to develop a Health promoting Schools

1. Engaging health and educational officials, teachers, students, parents and community leaders in efforts to promote health in schools
2. Providing a safe, healthy environment, both physical and psychosocial
3. Providing effective skills based health education and life skills
4. Providing access to health services
5. Implementing school policies and practices that support health
6. Striving to improve the health of the community

How to promote Healthy Workplace in your area?

A healthy workplace means more than just a safe and healthy physical workspace. Employers must also address the organizational culture and the health practices of employees. This approach will provide an opportunity to resolve basic health problems by creating synergies between occupational health, health protection and promotion, human resources management, sustainable

human development, and environmental protection. Timely and strict implementation will help to prevent and control lifestyle related NCDs in industrial settings.

Fig 2: Broad Framework for Developing a Healthy Workplace

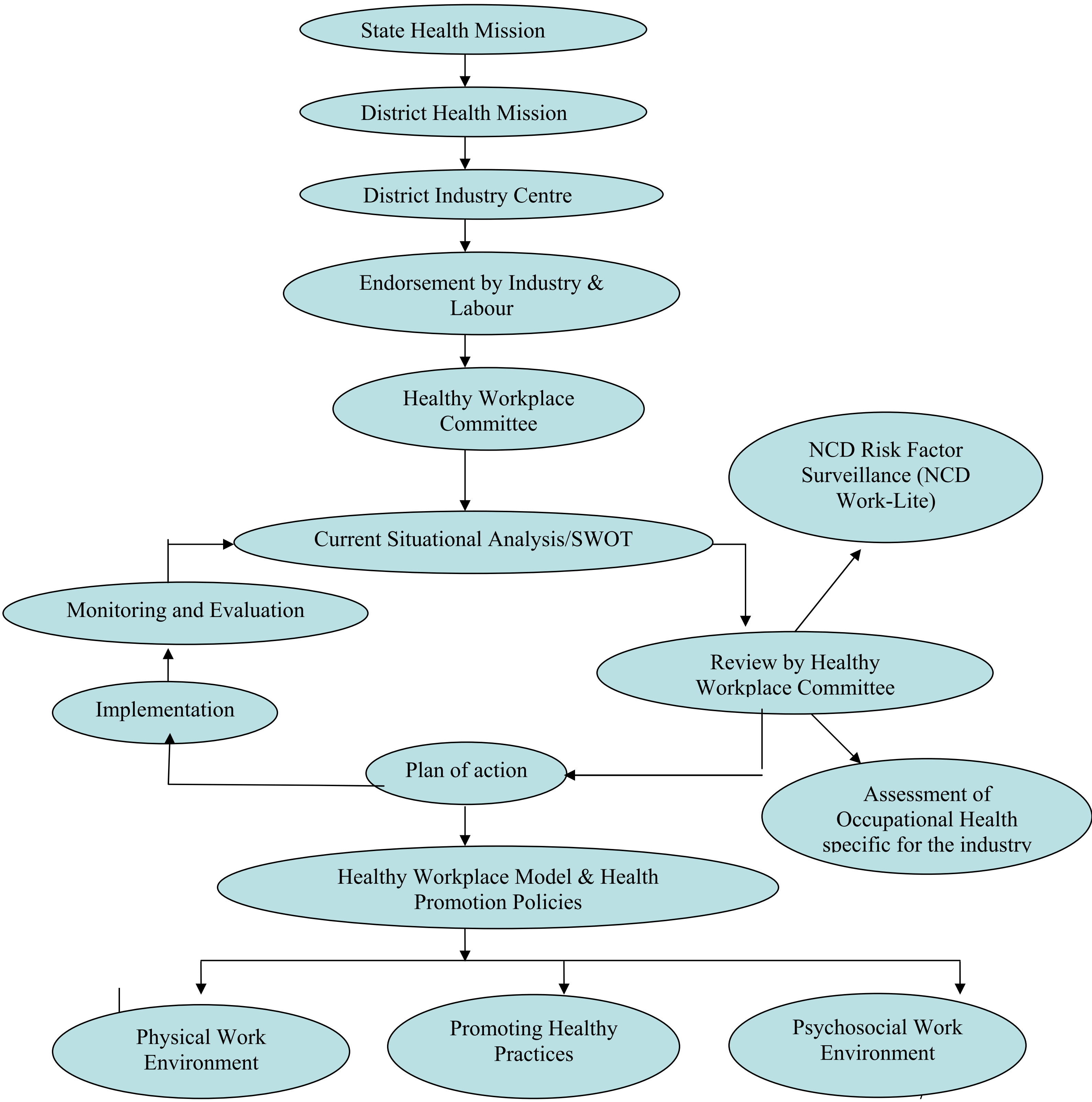

Why the workplace is ideal for NCD prevention?

- An easy, concentrated population to target
- Multiple levels of influence can be used – direct, such as the provision of healthy food, and indirect, such as creating a supportive environment
- Possible to link workplace health promotion to other programmes at the workplace, such as occupational safety programmes
- Concentration of lifestyle-related risk factors at the workplace
- The workplace has been recognized internationally as an important health promotion setting

As medical officer you should play a proactive role to initiate implementation of healthy workplace concept in your area with the help of paramedical workers, opinion leaders and community volunteers etc. The scheme to develop a healthy workplace is attached for your reference.

Section 3: RISK FACTORS OF NCDS

What gives rise to major NCDs (Diabetes, cardiovascular diseases, Stroke)?

Major NCDs are caused by a set of risk factors like unhealthy diet (low fruit and vegetable intake), physical inactivity, tobacco use, harmful use of alcohol and stress. High blood pressure, dyslipidemia (high levels of total cholesterol, LDL-cholesterol, and triglycerides and low level of HDL-Cholesterol) overweight/obesity (both generalized and central) are other physiological risk factors. Other putative but not well proven factors include air pollution, food preservatives, adulterants, artificial color and indoor smoke from solid fuels. Alcohol consumption, specifically binge drinking, leads to acute hypertension, stroke and in some individuals atrial fibrillation and cardiomyopathy.

Risk factors and level of NCD prevention and management

**Behavioral RF**	→	**Physiological RF**	→	**Disease Outcome**
• Unhealthy Diet<br>• Physical inactivity<br>• Tobacco<br>• Alcohol<br>• Stress		• BMI (Obesity)<br>• Hypertension<br>• Hyper-cholesterolemia<br>• High Blood sugar level		• Diabetes<br>• Heart disease<br>• Stroke<br>• Cancer<br>• Chronic respiratory disease
Primary Prevention<br>Health promotion		Secondary Prevention<br>Case management & HP		Tertiary Prevention<br>Case management

Some attributes of risk factors

- Risky lifestyle behaviors are responsible for the risk factors. Risk factors are cumulative and operate on a life course perspective. (i.e. they influence the risk throughout the life course. For example childhood obesity is a major risk factor for adult obesity and consequently diabetes and CVD).
- Normally, for all practical purposes it is seen that these risk factors occur together. A person who has high blood sugar levels may also have high blood pressure, dyslipidemia and central obesity.
- Risk factors operate in a continuum. This means that even within the normal ranges, people with higher level have higher risks. For example individuals with systolic blood pressure of 140 mmHg have a higher risk of CVD, stroke and future death than those with 120 mmHg even though both are within 'normal' range. This applies to all the risk factors of CVD and Stroke
- The risk factors are additive. This means cumulative small elevations of risk factors are much more harmful than isolated elevation of a single risk factors.

It is important to note that all these risk factors are amenable to modification through lifestyle changes. **In nutshell, today's risky behaviors are tomorrow's risk factors. Today's risk factors are tomorrow's disease**. **Thus, primary and secondary prevention of chronic diseases and their common risk factors provide the most sustainable and cost-effective approach to chronic disease prevention and control.**

Section 4: RISK ASSESSMENT AND MANAGEMENT

This section provides evidence-based guidelines on how to reduce the occurrence of first clinical events of coronary heart disease (CHD), cerebrovascular disease (CeVD) and peripheral vascular disease (PVD) in the population.

The evidence-based recommendations given in these guidelines provide guidance on which specific preventive actions to initiate, and with what degree of intensity. The accompanying World Health Organization/ International Society of Hypertension (WHO/ISH) risk prediction charts enable the estimation of total cardiovascular risk of people in the first category.

4.1. What are the goals of implementing these guidelines?

The goals are to prevent CHD, CeVD and PVD events by lowering cardiovascular risk.
The recommendations assist people to:

- Quit tobacco use, or reduce the amount smoked, or not just start the habit
- Make healthy food choices
- Be physically active
- Reduce body mass index, waist hip ratio/waist circumference
- Lower blood pressure
- Lower blood cholesterol and low density lipoprotein cholesterol (LDL-cholesterol)
- Control hyperglycaemia
- Take anti platelet therapy when necessary.

4.2. Who needs referral to a specialist facility?

Referral is required if there are clinical features suggestive of:

- Acute cardiovascular events such as: heart attack, angina, heart failure, arrhythmias, stroke, and transient ischemic attack.
- Secondary hypertension, malignant hypertension.
- Diabetes mellitus (newly diagnosed or uncontrolled).
- Established cardiovascular disease (newly diagnosed or if not assessed in a specialist facility).
- People needing medical therapy to quit smoking.

Once the condition of the above categories of people is assessed and stabilized, they can be followed up in a primary care facility based on the recommendations provided in these pocket guidelines. They will need periodic reassessment in specialty care.

4.3. When is grading cardiovascular risk using charts unnecessary for making treatment decisions?

Some individuals are at high cardiovascular risk because they have established cardiovascular disease or very high levels of individual risk factors. Risk stratification is not necessary for making treatment decisions for these individuals as they belong to the high risk category; all of them need intensive lifestyle interventions and appropriate drug therapy . They include people:

- with established cardiovascular disease
- without established CVD who have a total cholesterol ≥ 320 mg/dl or low-density lipoprotein (LDL) cholesterol ≥ 240 mg/dl or TC/HDL-C (total cholesterol/high density lipoprotein cholesterol) ratio >8
- without established CVD who have persistent raised blood pressure of

≥160/ ≥100 mmHg

- with renal failure or renal impairment.

4.4. Instructions for using WHO/ISH risk prediction charts

These WHO/ISH risk prediction charts indicate 10-year risk of a fatal or nonfatal major cardiovascular events (myocardial infarction or stroke), according to age, gender, blood pressure, smoking status, total blood cholesterol and presence or absence of diabetes mellitus. There are two sets of charts. One set can be used in settings where blood cholesterol can be measured. The other set is for settings in which blood cholesterol cannot be measured.

The charts provide approximate estimates of CVD risk in people who do not have established coronary heart disease, stroke or other atherosclerotic disease. They are useful as tools to help identify those at high cardiovascular risk, and to motivate persons, particularly to change behaviour and, when appropriate, to take antihypertensive, lipid-lowering drugs, and aspirin.

4.5. How do you use the charts to assess cardiovascular risk?

- If blood cholesterol can be measured, refer to chart 1.
- If blood cholesterol cannot be measured due to resource limitations, refer to chart 2.
- Before applying the chart to estimate the 10 year cardiovascular risk of an individual, the following information is necessary
  - Presence or absence of diabetes*
  - Gender
  - Smoker (*All current smokers and those who quit smoking less than 1 year before the assessment*) or non-smoker**
  - Age
  - Systolic blood pressure (SBP) ***
  - Total blood cholesterol.

* *A person who has diabetes is defined as someone taking insulin or oral hypoglycaemic drug(s), or with a fasting venous plasma glucose concentration ≥126 mg/dl or a postprandial (approximately 2 hours after a main meal) venous plasma glucose concentration ≥ 200 mg/dl on two separate occasions. For very low resource settings urine sugar test may be used to screen for diabetes if blood glucose assay is not feasible. If urine sugar test is positive a confirmatory blood glucose test needs to be arranged to diagnose diabetes mellitus.*

** *All current smokers and those who quit smoking less than 1 year before the assessment are considered smokers for assessing cardiovascular risk.*

*** *Systolic blood pressure, taken as the mean of two readings on each of two occasions, is sufficient for assessing risk but not for establishing a pre-treatment baseline.*

Once the above information is available proceed to estimate the 10-year cardiovascular risk as follows:

**Step 1**	Select the appropriate chart depending on the presence or absence of diabetes
**Step 2**	Select male or female tables
**Step 3**	Select smoker or non smoker boxes
**Step 4**	Select age group box (if age is 50-59 years select 50, if 60-69 years select 60 etc)
**Step 5**	Within this box find the nearest cell where the individual's systolic blood pressure (mm Hg) and total blood cholesterol level (mg/dl) cross. The colour of this cell determines the 10 year cardiovascular risk.

4.6. WHO / ISH Risk prediction CHARTS

Chart 1: 10 year risk of a fatal or non fatal cardiovascular event by gender, age, systolic blood pressure, total blood cholesterol, smoking status and presence or absence of diabetes mellitus

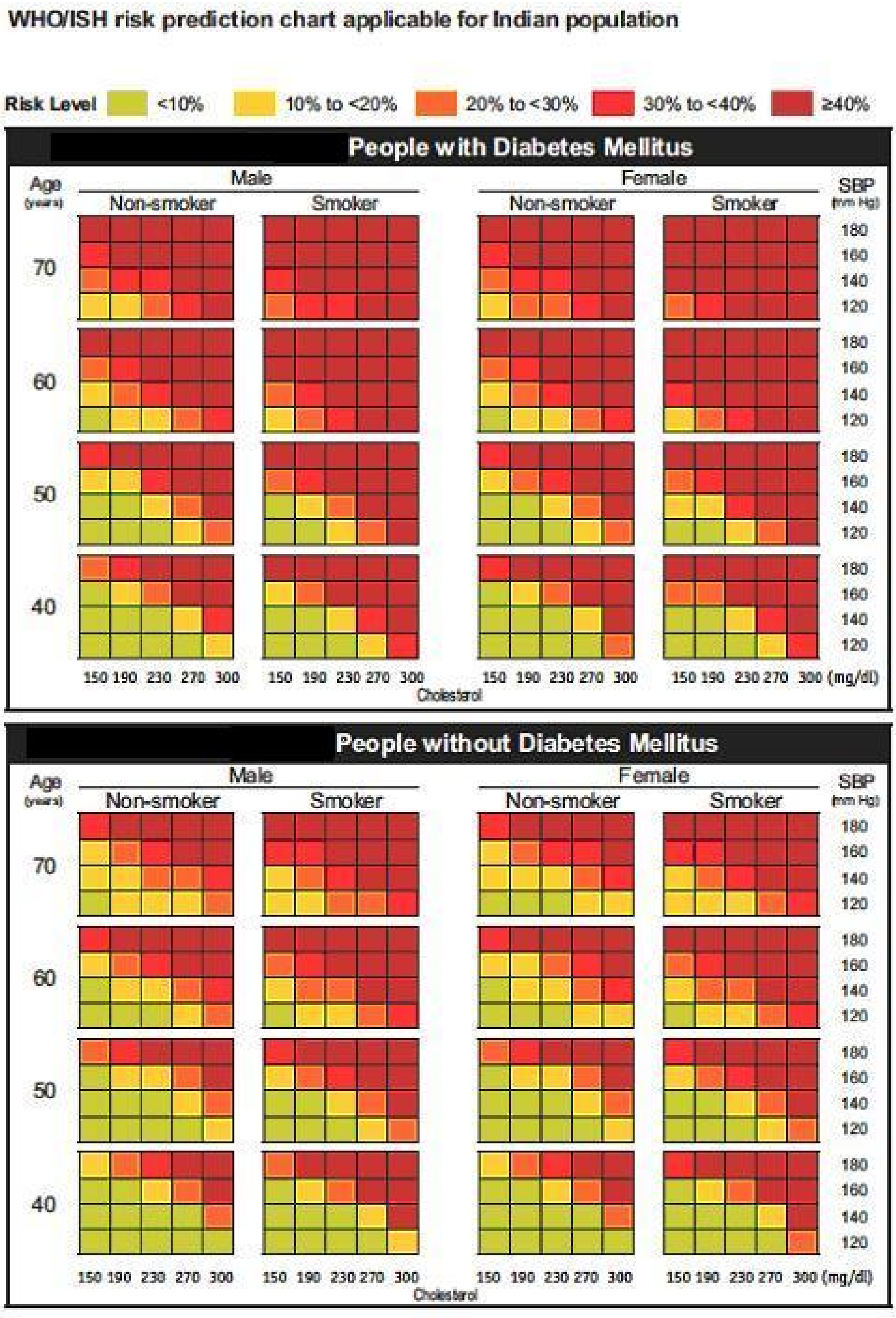

Chart 2: 10 year risk of a fatal or non fatal cardiovascular event by gender, age, systolic blood pressure, smoking status and presence or absence of diabetes mellitus

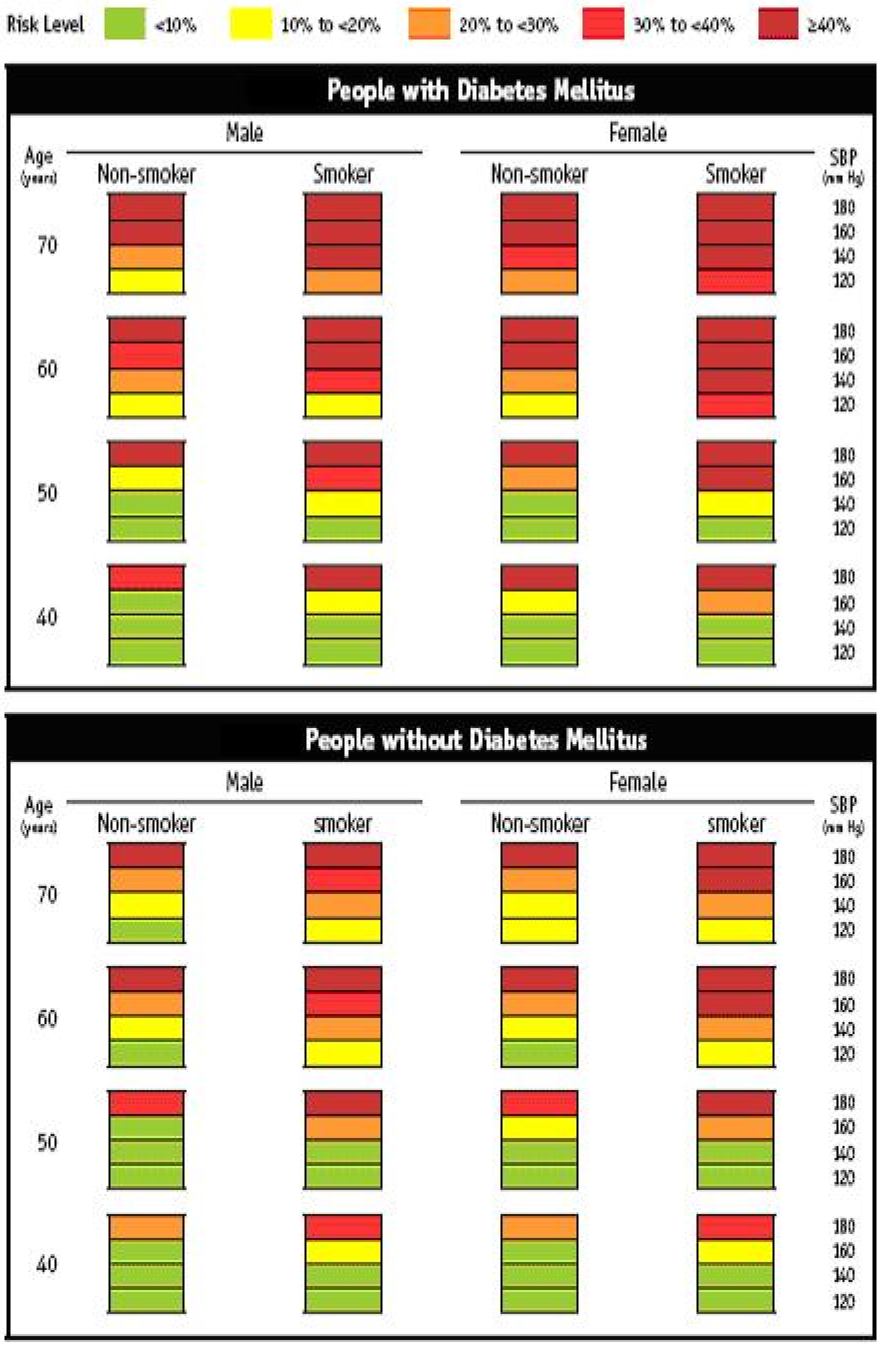

4.7. Practice Points

Please note that CVD risk may be higher than indicated by the charts in the presence of the following:

- ■■ Already on antihypertensive therapy
- ■■ Premature menopause
- ■■ Approaching the next age category or systolic blood pressure category
- ■■ Obesity (including central obesity)
- ■■ Sedentary lifestyle
- ■■ Family history of premature CHD or stroke in first degree relative (male <55 years, female < 65 years)
- ■■ Raised triglyceride level (≥150 mg/dl)
- ■■ Low HDL cholesterol level (≤40mg/dl in males, ≤ 50 mg/dl in females)
- ■■ Fasting glycaemia, or impaired glucose tolerance
- ■■ Microalbuminuria
- ■■ Socioeconomic deprivation.

4.8. Recommendations for prevention of cardiovascular disease in people with cardiovascular risk factors (according to individual total risk)

10 year risk of cardiovascular event	Risk classification	Intervention
**Risk <10%**	**LOW RISK**	Low risk does not mean "no" risk.<br>Conservative management focusing on lifestyle interventions is suggested.<br>Risk assessed after 5 years unless significant change in health status.
**Risk 10% to <20%**	**MODERATE RISK**	Monitor risk profile every 2 years.
**Risk 20% to <30%**	**HIGH RISK**	Monitor risk profile yearly.
**Risk ≥30%**	**VERY HIGH RISK**	Individuals in this category are at very high risk of fatal or non-fatal vascular events.<br>Monitor risk profile every 3–6 months.

Note: Reassess a person after six months of lifestyle modification if there is isolated elevation of single risk factor.

4.9. LIFESTYLE MODIFICATION

a. DIET

- Increase intake of green leafy vegetables and fresh fruits.
- Consume less salt; avoid adding/sprinkling salt to cooked and uncooked food.
- Preparations which are high in salt and need to be moderated are: Pickles, chutneys, sauces and ketchups, papads, chips and salted biscuits, cheese and salted butter, bakery products and dried salted fish.

- Restrict all forms of sugar free and refined carbohydrates for example biscuits, breads, naan, kulchas, cakes, mathris etc.
- Steamed and boiled food should be preferred over fried food.
- Have fresh lime water instead of carbonated drinks.
- Avoid eating fast/junk foods and aerated drinks. Instead of fried snacks, eat a fruit.
- In practice, it is best to use mixture of oils. Either buy different oils every month or cook different food items in different oils.
- Oils which can be mixed and matched are mustard oil, soya bean oil, groundnut oil, olive oil, sesame oil, and sunflower oil.
- Ghee, vanaspati, margarine, butter and coconut oil are harmful and should be moderated.
- If you are a non vegetarian, try to take more of fish and chicken. They should not be fried. Red meat should be consumed in small quantities and less frequently.

b. PHYSICAL ACTIVITY#

- Physical activity is a key determinant of energy expenditure.
- Regular exercise is important for promoting weight control or weight loss.
- Exercise regularly (moderate to vigorous) for 5-7 days per week; start slowly and work up gradually
    - At least 30 minutes (accumulated) of physical activities per day for cardiovascular disease protection.
    - 45 minutes/ day (accumulated) for fitness.
    - 60 minutes/ day (accumulated) for weight reduction.
- Discourage spending long hours in front of TV.
- Encourage outdoor activities like cycling, gardening etc.
- **Yoga**: A holistic life style which includes Asanas and all other components of healthy life style like low fat vegetarian diet (Satvik diet), stress management, tobacco avoidance and physical exercise. They have the potential for primary and secondary prevention of heart disease.

c. WEIGHT CONTROL

All individuals who are overweight or obese should be encouraged to lose weight through a combination of a low calorie diet and dynamic physical activity.
Overweight or obesity is assessed by measuring body mass index (BMI), which is calculated as weight in kg/height in meter^2. For Indian population 18.5 to 22.9 BMI is normal, 23 to 24.9 is considered as overweight and BMI of ≥25 is considered as obesity. Waist circumference is also an important measurement of central obesity and it should be <90 cm for men and <80 cm for women. Another measure of central obesity is Waist Hip Ratio (WHR). Normal WHR is <0.85 for women and <0.95 for men.

# Patients with uncontrolled hypertension (≥200/≥110), uncontrolled diabetes (FBS ≥250 mg/dl), diminished vision due to diabetic/hypertensive retinopathy or for other reasons, recent myocardial infarction/unstable angina or stroke (within 6 weeks), and with uncontrolled angina (class III or more) are not advised to go for physical exercise.

d. TOBACCO CESSATION

All non-smokers should be encouraged not to start smoking.
All smokers should be strongly encouraged to quit smoking by a health professional through Lifestyle modification including YOGA and supported in their efforts to do so.
It is suggested that those who use other forms of tobacco be advised to stop. The following flow chart depicts the protocol for counseling on tobacco cessation using the 5 steps -5 A approach.

PROTOCOL FOR COUNSELING ON CESSATION OF TOBACCO USE
(5 STEPS– 5As)

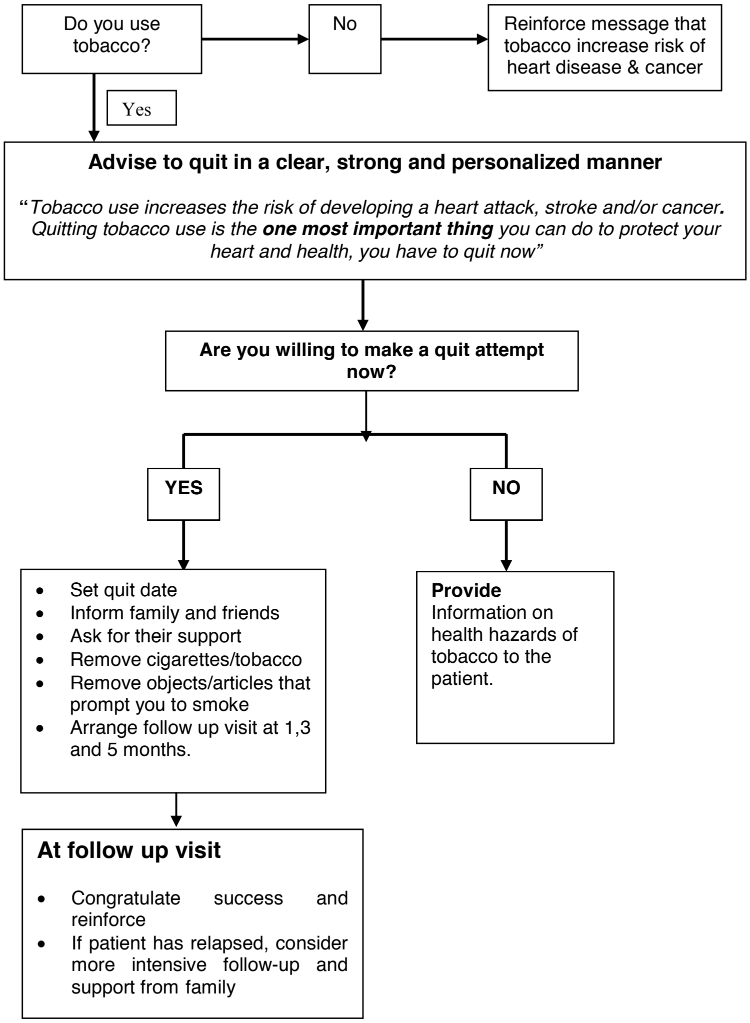

USE 5 STEPS- 5 As- ASK, ADVISE, ASSESS, ASSIST, ARRANGE.

e. ALCOHOL INTAKE

All individuals should avoid alcohol as far as possible.

4.10. PHARMACOTHERAPY

10year risk of cardiovascular event	Risk classification	Anti-hypertensive drugs	Lipid lowering drugs	Antiplatelet drugs
**Risk <10%**	**LOW RISK**	• Persistent blood pressure ≥140/90 mmHg should continue non pharmacologic therapy like lifestyle strategies<br>• Total cardiovascular risk reassessed every five years	• individuals with total cholesterol at or above 320 mg/dl<br>• To follow a non pharmacologic therapy i.e., lifestyle modification	• Aspirin should *not* be given to individuals in this low-risk category.
**Risk 10% to <20%**	**MODERAT E RISK**	• Persistent blood pressure ≥140/90 mmHg should continue non pharmacologic therapy like lifestyle strategies<br>• Total cardiovascular risk reassessed every two years	• individuals with total cholesterol at or above 320 mg/dl<br>• To follow a non pharmacologic therapy i.e., lifestyle modification	• Aspirin should *not* be given to individuals in this risk category.
**Risk 20% to <30%**	**HIGH RISK**	• Persistent blood pressure ≥140/90 mmHg should continue non pharmacologic therapy like lifestyle strategies<br>• Total cardiovascular risk reassessed every year<br>• First line antihypertensive therapy may be considered.	Adults >40 years with persistently high serum cholesterol (>200 mg/dl) and/or LDL cholesterol >120 mg/dl, despite a lipid-lowering diet and other lifestyle measures for a year, should be given a statin.	• Aspirin should *not* be given to individuals in this risk category.
**Risk ≥30%**	**VERY HIGH RISK**	• Persistent blood pressure ≥130/80 mmHg should continue non pharmacologic therapy like lifestyle strategies<br>• Total cardiovascular risk reassessed six months<br>• First line antihypertensive therapy may be considered.	Adults >40 years with persistently high serum cholesterol (>200 mg/dl) and/or LDL cholesterol >120 mg/dl, despite a lipid-lowering diet and other lifestyle measures for a year, should be given a stain.	• Aspirin should be given to individuals in this risk category.

Note: Reassess a person after six months of lifestyle modification if there is isolated elevation of single risk factor.

Section 5: MANAGEMENT GUIDELINES

5. 1. PREVENTION AND MANAGEMENT OF DIABETES

What is diabetes?

Diabetes is a disease in which the body does not produce or properly use the hormone insulin. The body needs insulin to convert sugar, starches and other foods into energy. Impairment of insulin secretion and action in the body leads to abnormally elevated levels of glucose in blood, a condition classically termed as diabetes.

What are the different "types" of diabetes?

Diabetes is classified into three types namely Type 1 Diabetes, Type 2 Diabetes and gestational diabetes. A description of each of these types is give below while guidelines for management elaborated in the following sections are specific to type 2 diabetes.

Type 1 diabetes (T1DM): usually occurs in younger people, children and adolescents. The diagnosis of T1DM can be made throughout childhood but it is more likely below 15 yrs of age. The onset is usually acute and severe and insulin is required for survival Type 1 diabetes results from autoimmune destruction of the beta cells in the pancreatic islets. Family history of diabetes is rare in T1DM. Presence of features of associated autoimmunity (autoimmune disorders, vitiligo) and absence of obesity and acanthosis nigricans are characteristics of T1DM. In addition, urine of T1DM patients with uncontrolled hyperglycemia is positive for ketone bodies.

Type 2 diabetes (T2DM): is the commonest type of diabetes. It usually occurs after the age of forty years but occurs frequently even at lower age among Indians. T2DM was previously known as non-insulin dependant diabetes mellitus. The onset is usually insidious and may be mild to severe. The family history is usually positive and strong. Obesity, metabolic syndrome and acanthosis nigricans are usually seen in these patients while there is no evidence of autoimmunity. Further, there is no insulin dependence till late in the course of illness.

When is a person at high risk for diabetes?

1. If he/she is of above the age of 30 years
2. If he/she is overweight (BMI is more than 23kg/m^2).
3. If he/she is physically inactive, that is, he or she exercises less than 3 times a week.
4. If he/she has high blood pressure.

5. If he/she has impaired fasting glucose or impaired glucose tolerance.
6. If his/her triglyceride and/or cholesterol levels are higher than normal.
7. If his/her parents/siblings or grandparents have or had diabetes.
8. If she delivered a baby whose birth weight was 4 kgs or more.
9. If she has had diabetes or even mild elevation of blood sugars during pregnancy.

When to suspect diabetes?

- Symptoms of uncontrolled hyperglycemia: excess thirst, excess urination, excess hunger with loss of weight
- Frequent infections
- Non-healing wounds
- Unexplained lassitude
- Fatigue
- Impotence in men

Criteria for diagnosing T2DM

The criteria for diagnosing T2DM, as defined by the World Health Organization in 1999, are depicted in table-5.1.1.

Table-5.1.1: Criteria for diagnosis of T2DM using venous blood samples*

	Fasting Glucose (mg/dl)	2-hour Post-Glucose Load (mg/dl)
Diabetes Mellitus	>=126	>=200
Impaired Glucose Tolerance	< 110	>140 to <200
Impaired Fasting Glucose	>=110 to <126	

**WHO Definition 1999*

Management of Diabetes

Management of T2DM should be initiated as soon as diagnosis is established even if the patient is asymptomatic. Initial assessment and management of the patients has to be carried out at Community Health Centre (CHC) level or at secondary care level. Management of T2DM comprises initial assessment, initial management and follow-up visits. Each of these components is elaborated here.

1. *Initial assessment* of individuals suspected of having T1DM need to be subjected to risk assessment which include:
   - History and physical examination;
   - Assessment of blood glucose level;

- Presence of CVD risk factors (lipid profile); and
- End-organ damage (urine for protein/ ECG/ fundus examination)

Assessment of history and physical examination of the patient is elaborated in table-5.1.2.

**Table-5.1.2 Initial Assessment of Diabetic Patients**	
**History (Ask for)**	**Physical Examination (Look for)**
Symptoms of hyperglycaemia	Weight
Duration since onset of symptoms	Body Mass Index
Precipitating factors such as recent infections, stress, change in dietary habits or physical activity levels	Waist circumference, Waist-hip ratio
Symptoms of Micro- and Macro-vascular Complications: visual disturbances, edema, breathlessness, angina, intermittent claudication, numbness, paraesthesiae	Acanthosis nigricans *
Hypertension, pre-existing cardiovascular diseases	Blood pressure
Drug history	Peripheral pulses
Diet	Feet: calluses, ulcers, prominent veins, edema, injuries
Physical Activity: type, frequency	Fundus examination#
Family History	Cardiovascular system
-Diabetes and complications<br>-Age at onset<br>-Cardiovascular disease	Peripheral nervous system<br>Thyroid
**Acanthosis nigricans is a brown to black, poorly defined, velvety hyperpigmentation of the skin, usually present in the posterior and lateral folds of the neck, the axilla, groin, umbilicus, and other areas. This occurs due to insulin spillover (from excessive production due to obesity or insulin resistance) into the skin which results in its abnormal growth, and the stimulation of color producing cells. The most common cause would be insulin resistance, usually from type-2 diabetes mellitus.*<br><br>*#Details of Fundus examination are provided in a later section*	

2. *Initial management* include:
   - Pharmacotherapy for the management of hyperglycaemia and any other co-morbid conditions e.g. high blood pressure, dyslipidemia etc.;
   - Therapeutic lifestyle management (please refer the earlier section); and
   - Diabetes patient Education and counseling by dietician

T2DM: ***Principles of Management***

Lifestyle management (diet and physical activity) accompanied by drug therapy or insulin are the corner stone of diabetes management. Apart from this other concurrent complications should be addressed. The basic principles in the management of type-2 diabetes are:

- Modify Lifestyle: diet and physical activity
- Reduce Insulin Resistance through reduction in weight, specifically reduction of fat mass
- Pharmacological treatment (if inadequate control):
    - *Sulfonylureas/ Metformin*
- Treatment for high blood pressure:
    - *ACE-Inhibitors, Calcium channel blockers such as amlodipine and diuretics such as hydrochlorothiazide*
    - *For details refer the section on hypertension*
- Lipid control with statins

The targets of control in Diabetes management in depicted in the Box 5.1.1

Box 5.1.1: Ideal Targets of control in the management of Diabetes

Fasting blood glucose	: 80-110 mg/dl
Post meal blood glucose	: 120-140 mg/dl
HbA_{1C}	: < 7%
Total cholesterol	: < 180 mg/dl
LDL-chol	: < 100 mg/dl
HDL chol	: > 45 mg/dl
Blood pressure	: < 130/80 mmHg
Serum TG	: <150 mg/dl

Source: ICMR

Note: The targets for diabetic population are lower than the non-diabetics

Box 5.1.2: Glycosylated haemoglobin (HbA_{1C})

A fraction of hemoglobin in the RBCs is found to be in a glycosylated form i.e. has glucose attached to it. The HbA1c level is proportional to average blood glucose concentration over the previous two to three months and therefore is an excellent indicator of how well the patient has managed his/her diabetes over the last four weeks to three months. Glycated hemoglobin is recommended for monitoring blood sugar control in diabetic patients.

American Diabetes Association (ADA) recommends an HbA1c goal of less than 7% for people with diabetes in general.

Pharmacotherapy

- BIGUANIDES (Metformin)

  Mechanism of Action: Insulin sensitizer

  Dose: The dose of metformin varies from 250mg to 2000mg/day. Since patients may complain of nausea and gastric irritation, the dose can be administered after a major meal. Dose of metformin can be titrated based on blood glucose monitoring at intervals of 2-4 weeks. Currently the preferred approach is to start the patient on metformin and increase the dose to at least 1g/day. If despite this dose, optimum glucose control is not achieved, a suphonylurea should be added (see box-7 for targets of control.

  Advantages

  - No weight gain; some patients may experience weight loss. Hence metformin is useful in large majority of patients who are overweight
  - No hypoglycaemia
  - For monotherapy in obese patients
  - Can be combined with other anti-hyperglycaemic agents including insulin

  Contraindications

  - renal (Creatinine ≥ 1.5mg% in men; Creatinine ≥ 1.4mg% in women) / hepatic disease
  - cardiac / respiratory insufficiency; other hypoxic condition
  - severe infections
  - alcohol abuse
  - history of lactic acidosis
  - Use of I/V radiographic contrast media
  - Pregnancy
  - Temporarily withhold: surgery, acute illness
  - **Caution:** Phenformin is a banned drug and is not recommended.

- SULPHONYLUREAS (Glibenclamide)
    - The dose of glibenclamide varies from 2.5-20mg/day given in one or two doses. The dose can be titrated based on blood glucose monitoring at intervals of 1-2 weeks
    - General rule: glucose lowering effect plateaus after half-maximal recommended dose
    - APPROVED INDICATIONS: monotherapy; in combination with metformin and insulin
    - Caution: Hypoglycemia can occur most likely to effect among elderly, those with worsening renal function and among those with irregular meal schedules

General Guidelines for using oral anti-diabetic agents:

The treatment should be individualized and the points mentioned below are only broad based Guidelines. The necessity of diet, exercise and life style modifications needs to be emphasized; in some cases these measures alone would suffice. When pharmacological treatment becomes necessary, the following points may be considered:

(i) <u>Non-obese people with type 2 diabetes:</u>

- In non-obese people with diabetes, start with a sulphonylurea / meglitinide or glitazone. If even after two to four weeks of initiation of treatment, symptoms still persist or blood sugar is not sufficiently controlled then a drug from another group like metformin can be added. If the initial blood sugar levels are very high, the symptoms are very severe or acute complications like ketosis are present, insulin has to be considered for treatment even at the onset, for a brief period.

- If the initial assessment shows presence of complications like diabetic retinopathy or nephropathy, this indicates a long period of undiagnosed diabetes and insulin therapy on a continuous basis should be considered.

(ii) <u>Obese people with type 2 diabetes:</u>

- In obese people with diabetes, the starting drug is ideally metformin.
- Similar Guidelines as mentioned above can be used to achieve good metabolic control with addition of other drugs like sulphonylureas/ meglitinides or glitazones and/ or insulin.

(iii) <u>Lean people with type 2 diabetes:</u>

- In India, many subjects with type 2 diabetes are lean or low body weight (BMI $<18.5 kg/m^2$). In these people with diabetes, metformin is better avoided and the use

of glitazones and sulphonylureas may be considered as first line of management. Quite often, such people with diabetes may require insulin for better control.

- With increasing duration of diabetes, most oral anti-diabetic agents tend to be less effective and hence poly-pharmacy becomes inevitable, with use of drugs from multiple classes. However, insulin use should not be delayed and, if and when necessary, insulin should be introduced for tight glycemic control.

Combination of oral drugs with insulin

When the glycemic control is not achieved with the maximum dose of an oral agent/combination therapy, this is called "secondary failure to oral hypoglycemic agents (OHA).

It has been the experience of most physicians in India that combination of oral drugs and insulin helps to achieve good control of diabetes. While using combination therapy, the oral drugs may be continued in optimal doses, while intermediate acting/long acting/short acting insulin is added either at bed time or in the morning depending on the blood sugar profile of person with diabetes. However, if indicated, one should not hesitate to use insulin in multiple doses to achieve tight metabolic control.

When to recommend hospitalization

- Uncontrolled infections;
- Severe cellulitis,
- Unresponsive UTI or other deep seated infections including bad diabetic foot needing intravenous antibiotics,
- Recurrent UTI not responding to oral antibiotics,
- Presence of ketones in urine

Diabetes patient education and diet counseling

Patient education on diabetes management and life style modifications is the corner stone of effective diabetes control and management and prevention of complications**.** If a 'diabetes educator' is available then the patients and their families must visit him/her. At PHC level, nurses/multipurpose health workers can be trained to undertake this activity. At sub-district and district level hospital, dietician/counselor and nurses can under take diabetes patient education. Counseling on diet need to be provided by a trained dietician at district and sub-district level hospital. Patient education topics that can be covered in the initial visit and follow-up visits are depicted in the table-5.1.3.

Table-5.1.3: Patient education topics to be covered in the initial and follow-up visits	
**Initial Visits**	**Follow-up Visits**
What is Diabetes?	Importance of Glycaemic Control
Why does it occur?	Prevention of Complications
Lifestyle measures: Diet, Exercise	Foot Care (see box-5.1.3)
Detailed lifestyle advice	Newer modalities of treatment
Use of Oral Drugs	Marriage Counseling
Advice on identifying signs and symptoms of hypoglycaemia and hyperglycaemia and their management	Pre-conceptional counseling regarding the importance of good glucose control prior to pregnancy
Patient should be informed about the importance of factors other than glucose control: cholesterol, blood pressure, stopping smoking,/tobacco, etc	

Box 5.1.3: Foot Examination

Foot care advice to the patients

Inspect your feet daily for cracks, blisters, infections, and injuries. You may be able to see a problem before you feel it. If you can't see the bottoms of your feet easily, use a mirror. A magnifying glass also may help you see better. If you can't check your own feet, have someone else do it for you.

Cleanse your feet daily as you bathe or shower, using warm water and mild soap. Dry your feet with a soft towel making sure to dry between the toes. Don't use hot water. You may burn your skin as you may not be able to feel the hotness of the water.

Moisturize dry skin by using oil. If it causes redness or irritation, discontinue it's use and inform your doctor. If you are currently using a cream or lotion that keeps your skin soft and free of cracks, continue using it.

Clip toenails straight across. Use a nail cutter; don't use a scissor and also smooth down the edges. If you can't easily reach your feet for have thick nails, have someone experienced trim your nails.

Always wear something on your feet (socks, slippers, shoes) to protect from injury - even in your house.

Choose soft good shoes. Let them be a size bigger that what you feel is appropriate. Wear socks made of cotton or wool (in winter).

Treat minor breaks in the skin promptly. Cleanse the area with soap and water, dry, and cover with clean gauze. Observe for signs of infection such as redness, swelling, warmth, pain or drainage. Don't put weight on the foot that has an injury.

See your doctor to check your feet during your regular visits for diabetes care. Take off your shoes and socks at every visit.

For more information and visual guidance visit
http://www.healthy-india.org/preventdiabetes5.asp

3. *Follow-up visits*

   Annual assessment of the patients has to be carried out at CHC/secondary care level for follow-up of blood glucose, urinary microalbuminuria, fundus examination, blood lipids, creatinine, feet examination and patient education. Primary care physicians need to follow up the diabetic patients regularly for compliance with medicines, lifestyle management, blood glucose control, blood pressure control and control of other risk factors.

Eye Care in diabetes

The Retina/fundus of all diabetes patients need to be checked at least once a year by a trained ophthalmologist even if there are no eye symptoms and the vision is 6/6. The patient needs to be accordingly referred for the same to the CHC, where ophthalmologist is available. Early retinal problems don't show up as visual symptoms and a good vision should not mean that a fundus examination is not required.

Diabetes can damage blood vessels throughout our bodies. The vessels in the eyes seem especially vulnerable to damage. In the early stages of retinopathy, fluid can leak from small blood vessels in the retina. If this leaking occurs in the macula, then objects may appear blurry. However early damage can be diagnose through a retina/fundus examination and the blood vessels can be sealed with laser and vision can be saved and preserved.

Proliferative retinopathy is an advanced form of retinopathy. Proliferative retinopathy occurs when abnormal blood vessels grow on the retina and sometimes into other parts of the eye. If these vessels bleed into the vitreous - the clear fluid in the centre of the eye, light can't reach the retina and vision can become cloudy. The blood may be slowly reabsorbed and vision can return to normal but if the bleeding continues, vision may be cloudy until the problem is treated.

Tissue can also grow along with the abnormal blood vessels, distorting vision or making objects appear blurry. Over time, the tissue can shrink, pulling the retina away from its base.

If the blood doesn't reabsorb or if the tissue affects vision, the vitreous may need to be surgically removed to avoid loss of vision. All these problems of the eyes can be prevented if prevention is started early.

Checklist for preventing of diabetes complications

Every 3-6 months the patient should have a physical review by the physician. Checklist for the follow-up is as follows:

1. Test blood sugar levels
2. Test glycosylated haemoglobin levels(HbA_{1C}) (if facilities are readily available)
3. Examine feet for sensations and circulation; Also for calluses, dryness, sores, infections, injuries
4. Check blood pressure.
5. Help the patient to give up tobacco, if he/she continues to use tobacco
6. Reinforce of life style measures- increase physical activity levels and improve diet (please refer the earlier section on therapeutic lifestyle management).

Preconception counselling

Counselling on pregnancy must start before conception. All women with diabetes must know that they should not conceive till their blood glucose is well controlled for at least 2-3 months before conception as ascertained by HbA_{1C}. Hyperglycemia at conception increases the risk of complications during pregnancy as well as congenital defects in the foetus. A summary of services for diabetes management, appropriate at each levels of care, is depicted in the table 5.1.4:

**Table-5.1.4: Management of Diabetes at each levels of care**			
Services	Levels of Care		
	Secondary care level	CHCs	PHCs
Screening for Diabetes	√	√	√
History and Physical Examination	√	√	√
Initial Assessment	√	√	X
Diabetic Patient Education	√	√	√
Pharmacotherapy			
Initiation	√	√	X
Follow-up	√	√	√
Foot care	√	√	√
Eye care	√	√	X
Annual Assessment	√	√	X
Pre-conception Counseling	√	√	√
Marriage Counseling	√	√	√

5.2. PREVENTION AND MANAGEMENT OF HYPERTENSION

Introduction

Abnormally elevated blood pressure is a pathological condition which increases the work load on the heart. This condition is termed as high blood pressure or hypertension. Based on the etiology, high blood pressure is of two types:

Primary/essential: Primary or "essential" hypertension has no known cause, however many of the above said lifestyle factors are associated with this condition. This constitutes majority of the high blood pressure in the world today**.**

Secondary: Secondary hypertension is caused by some other medical conditions/problem or the use of certain medications. Secondary hypertension is seen only in very few individuals in the community. The causes of secondary hypertension include: kidney diseases: reno-vascular disease and chronic renal disease, endocrine disorders: hyperthyroidism, cushing's syndrome and pheocromocytoma, sleep disorders, coarctation of the aorta and non specific aorto-arteritis. Some of these causes are often curable, and many others treatable.

Criteria for diagnosing high blood pressure

The table-5.2.1 provides a classification of blood pressure for adults ages 18 and older. The classification is based on consistent elevation during two or more properly measured BP readings in sitting position.

Table-5.2.1: Criteria for diagnosing high blood pressure

Category	Systolic	Diastolic
Normal	Less than 120	Less than 80
Pre-hypertension	120-139	80-89
**High Blood Pressure**		
Stage 1	140-159	90-99
Stage 2	160 or higher	100 or higher

Source: JNC VII classification

MANAGEMENT OF HYPERTENSION:

The Risk assessment should cover:

Assessment of medical history
Physical Examination

Laboratory Investigation

Assessment of medical history:

Ask for:

a. Risk factors

  - o Lack of physical activity (or sedentary lifestyle).
  - o Obesity or being overweight
  - o Abdominal obesity
  - o High sodium intake/high salt intake
  - o Excess alcohol consumption

b. Family history
c. Symptoms of consequences of hypertension
d. Frequent intake of pain relieving drugs (NSAIDS)
e. Steroid intake for asthma
f. Breathing difficulty particularly on exertion
g. Swelling of feet
h. Urinary difficulties, history of passing stones in the past

Physical examination:

Physical examination should include

a. BP measurement at least in one upper and one lower limb (Refer Appendix- for the procedure)
b. Measurement of Body weight and height to obtain BMI
c. Measurement of Waist circumference
d. Palpating all peripheral pulses
e. Auscultation for bruit (renal, carotid, abdominal and others)
f. Eye evaluation if ophthalmology facility is available

Laboratory Tests:

Essential:

- Blood Sugar
- Urine analysis for proteinuria

Desirable: (at CHC/sub-district/district level hospitals depending upon the available facilities for laboratory investigations)

- Haemogram,
- Serum creatinine
- Serum sodium and potassium levels
- Lipid profile
- Complete Urine analysis
- Electrocardiogram(ECG)
- X-Ray chest

Based on risk assessment, the management of high blood pressure cases can be initiated. The management should include the following:

- *Therapeutic life-style management (refer to section on lifestyle modification)*
- *Drug Therapy*

Drug Therapy

Whether a person requires medicines for his high blood pressure and **which** medicine is best for the patient would depend on:

- The blood pressure reading
- Whether the high blood pressure has already affected target organs in the body such as heart, kidneys, eyes and arteries
- Concurrent medical conditions such as diabetes, heart disease, kidney disease and other risk factors like use of tobacco, obesity and high blood fat levels(lipid profile) etc.
- Other considerations will be age, gender (male/female) and body weight.

Treatment Goals

Treatment Goals

Refer to the risk assessment section to manage hypertension.

1. Initial aim should be to obtain blood pressure level less than 130/85 mms of Hg
2. Ideally the aim should be to get to blood pressure levels of less than **120/80** without bothersome side-effects.
3. Don't accept blood pressure levels of **140/90** mms of Hg or more
4. Maintain healthy blood pressure throughout the person’s lives
5. Prevent and control risk factors which could give rise to high blood pressure.
6. Always make sure that risk factors **are controlled**.
7. Prevent and control risk factors which could increase risk of complications due to high blood pressure.

In the Indian context, **diuretics (hydrochchlorthiozide), calcium channel blockers (amlodipine) and ACE inhibitors (Enalapril)** are relatively cheap. Drug therapy should be started in individuals at the time of diagnosis if therey have blood pressure more than 160/100mmHg (despite non-pharmacological interventions) or if the blood pressure is more than

140/90 in diabetic subjects or end organ damage such as protinuria, high blood urea, ECG evidence of left ventricular hypertrophy, presence of heart diseases and evidence of retinopathy. In all other individuals life style modification should be tried for atleast six months before initiating drug therapy.

Medicines are tailored depending on the following factors

- Blood pressure level
- Patient characteristics (like age, body weight, occupation)
- Co-existing risk factors
- Type and extent of target organ damage
- Other associated diseases
- Affordability

It is better to start with calcium channel blockers (specifically if the person is older than 55 years) and ACE inhibitors if less than 55 years. Recheck the BP in 2 weeks. If BP is not under control adding diuretics (Hydrochlorothiazide 12.5 mg a day) may be helpful. Normally this should bring the BP under control. If the BP is not controlled by the combination of Amlodipine 10mg + Hydrochlorothiazide 25mg a day or Enalapril 10mg and Hydrochlorothiazide 25mg a day, a referral to a higher center may be necessary.

Life style advice is advocated for the first six month after the diagnosis of high BP in the following situations:

- If the BP is less than 160/100 mm of Hg
- There is no diabetes, co-existing heart disease stroke or peripheral vascular disease
- No evidence of LVH on ECG
- Absence of urinary proteinuria and
- Serum creatinine <1.6mg/dl

Table-5.2.2: Dosage and contraindications of Common anti-hypertensive medications			
**Drug**	**Dose**	**Contra-indications**	**Cautions**
ACE-Inhibitors	Enalapril: 5-40mg/day<br>Captopril: 25-150mg/ day<br>Ramipril: 2.5-20mg/day	Pregnancy<br>Renovascular diseases	Renal impairment<br>Peripheral vascular disease
Calcium-Channel blockers	Amlodipine: 2.5-10mg/day<br>Nifedipine (Sustained Release): 30-60mg/day	-	Painless swelling of the feet and ankles may occur leading to its withdrawal in a few patients
Thiazides	Hydrochlorothiazide: 12.5-50 mg/day	Gout	
Beta-Blockers	Metoprolol (Sustained Release): 50-200mg/day<br>Atenolol: 25-100mg/day	Asthma or chronic obstructive pulmonary disease, advanced<br>Heart block	Heart failure (symptomatic)<br>Peripheral vascular disease,<br>Diabetes (except with coronary heart disease)

The summary of services for hypertension management, appropriate at each levels of care is depicted in table -5.2.3.

Table-5.2.3: Management of Hypertension at different levels of care			
**Services**	**Levels of Care**		
	**Secondary care**	**CHCs**	**PHCs**
Screening for Hypertension	√	√	√
Initial Risk Assessment			
Assessment of Medical History	√	√	√
Physical Examination	√	√	√
Laboratory Investigation			
Essential	√	√	√
Desirable	√	√	
Therapeutic Lifestyle Management	√	√	√
Pharmacotherapy			
Initiation (Uncomplicated cases)	√	√	√
Initiation (Complicated cases)	√	√	√
Follow-up	√	√	√
Annual Assessment	√	√	√

5. 3. PREVENTION AND MANAGEMENT OF DYSLIPIDEMIA

What is Dyslipidemia?

Dyslipidemia is characterized by elevation of plasma cholesterol, triglycerides (TGs), or both, or a low high density lipoprotein (HDL) level that contributes to the development of atherosclerosis.

Cholesterol is a fatty substance (lipid) that is present in cell membranes and is a precursor of bile acids and steroid hormones. Cholesterol exists largely as LDS-cholesterol, VLDL cholesterol, and HDL cholesterol. Along with triglycerides they are tightly packed in a central core and surrounded by surface lipoprotein particles. Several large epidemiological investigations of human populations incriminate high levels of cholesterol as being atherogenic.

The positive relationship between serum cholesterol levels and the development of first or subsequent attacks of CHD is observed over a broad range of LDL-cholesterol levels; the higher the level, the greater the risk. Prospective study data suggest that the risk of CHD plateau at lower cholesterol levels, but this apparent plateau has disappeared in larger studies. In other words the risk of CHD is continuous at all levels of blood cholesterol therefore any definition is weighted based on risk-benefit and cost-effectiveness ratio and management is based on individualized risk of CHD.

Definition of High Blood Cholesterol

Table-5.3.1. Classification of blood lipid levels for therapeutic interpretation

Blood Lipids	Serum Level (mg/dl)
Total Cholesterol	
Desirable	<200
Borderline High	200-239
High	>240
Among Indians, based in the normal mean levels of cholesterol, it is preferred to have total cholesterol less than 160mg/dl	
LDL Cholesterol	
Optimal	<100
Near Optimal	100-129
Borderline High	130-159
High	160-189
Very High	≥190
Serum Triglycerides	
Normal	<150
Borderline High	150-199
High	200-499
Very High	≥500
Serum HDL Cholesterol	
Low	<40
High	≥60

The definition of abnormal blood cholesterol is as indicated in the table 5.3.1. These are based on the Adult Treatment Panel (ATP) III recommendations of the USA as there are no indigenous Indian prospective epidemiological data on risks of high blood cholesterol on CHD. Low HDL cholesterol is an important risk factor for CHD while high HDL cholesterol has a protective effect and is considered a negative risk factor.

MANAGEMENT OF HIGH BLOOD CHOLESTEROL

Management of high blood cholesterol would depend on the overall risk profile of an individual. The broad guidelines to assess management would be based on:

1. Ascertaining whether patient has established Cardiovascular Disease like previous heart attack/ stroke/angina/peripheral vascular disease.
2. is a diabetic or not
3. what other CVD risk factors (hypertension/smoking/age/obesity) are present
4. Blood sugar/ total cholesterol/ HDL/LDL cholesterol levels(where available)

Treatment Goals

PHC Level

1. All patients with established CVD or diabetes should be counseled about non pharmacology treatment and also initiated on statins (Atorvastatin /Simvastatin).
2. Other patients should be counseled about non pharmacology treatment

CHC Level

1. All patients with established CVD or diabetes should undergo lipid profile (where a detailed profile is not available total cholesterol should be done). They must be counseled about non pharmacology treatment and also initiated on statins (Atorvastatin /Simvastatin) to achieve a LDL cholesterol of less than 100 mg/dl or Total cholesterol of less than 200 g/dl. Patients not achieving these goals with statins alone or developing complications due to them should be referred to higher centre.
2. Other patients with 2 or more CVD risk factors should undergo lipid profile/ total cholesterol testing. Their risk level should be assessed by using **risk assessment chart** and **manage accordingly**.
3. Patients with one or no risk factors should be counseled about non pharmacology treatment only.

Drug Treatment

Several classes of hypolipidemic drugs are available. However, statins are by far the most widely used first line drugs as most of evidence of proven benefit of lowering cholesterol on CVD is based on trials using them.

**Table-5.3.2: Summary of Statins**	
Lipid effects	LDL cholesterol - ↓ 18–55%<br>HDL cholesterol - ↑ 5–15%<br>Triglycerides - ↓ 7–30%
Contraindications	Active or chronic liver disease
Major side/adverse effects	Myopathy, increased liver transaminases
Usual starting dose	Simvastatin - 20 mg<br>Atorvastatin - 10 mg

5.4 MANAGEMENT OF CAD

Definitions

Coronary artery disease is a condition in which there is an inadequate supply of blood and oxygen to a portion of the myocardium. It typically occurs when there is an imbalance between myocardial oxygen supply and demand. The most common cause of myocardial ischemia is atherosclerotic disease of an epicardial coronary artery or arteries (fig.5.4.1) sufficient to cause a regional reduction in myocardial blood flow and inadequate perfusion of the myocardium supplied by the involved coronary artery.

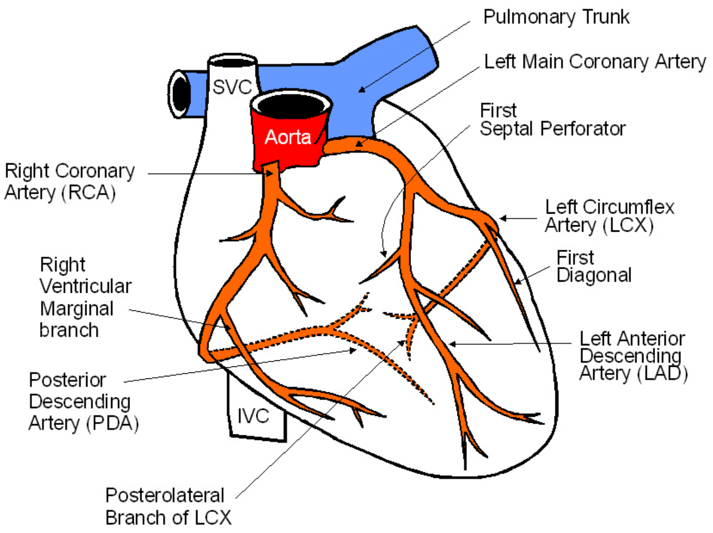

Fig 5.4.1: The coronary circulation representing specific arterial territory of the heart

- **Coronary Artery disease** (CAD): Fifty percent or more stenosis of epicardial coronary arteries.
- **Acute Coronary Syndrome** (ACS): A spectrum of clinical conditions from unstable angina to ST-elevation MI consequent to myocardial ischemia. Clinically, acute chest pain, typical in character, lasting more than 15 minutes. 'Typical' defined as retrosternal discomfort (heaviness), brought about or increased with exertion and reduced with rest or nitrates.
- **Unstable Angina** (UA): A clinical syndrome subset of ACS defined by ECG ST-segment depression or prominent T wave inversion and no elevation of cardiac biomarkers of necrosis (Troponins T/I or CPK_{MB}).
- **NSTEMI**: A clinical syndrome subset of ACS defined by ECG ST-segment depression or prominent T wave inversion and/or positive biomarkers of necrosis in the absence of ST-segment elevation.
- **STEMI:** A clinical syndrome subset of ACS characterized by ST-segment elevation or new onset LBBB due to myocardial necrosis.
- **Chronic Stable Angina:** Chronic manifestation of CAD described as retrosternal discomfort (heaviness), brought about or increased with exertion and reduced with rest or nitrates lasting less than 10 minutes.

The clinical spectrums of CAD are shown in the diagram below (fig.5.4.2).

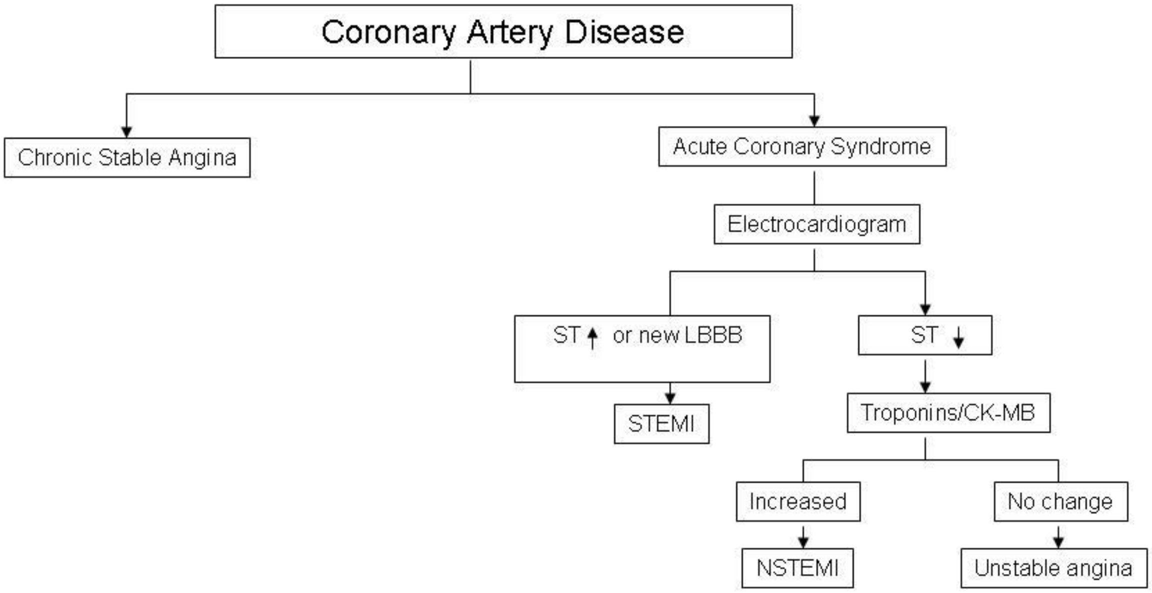

Fig.5.4.2: Flowchart showing the spectrum of CAD

Definition of myocardial infarction

MI was defined by a combination of two of three characteristics: typical symptoms (i.e., chest discomfort), enzyme rise and a typical ECG pattern. Pathologically, MI is defined as infarction of an area of the heart muscle, usually as a result of occlusion of an epicardial coronary artery.

Pathogenesis and pathophysiology

Coronary artery occlusion is a dynamic process from deposition of atherosclerotic plaque and partial occlusion to complete artery occlusion (fig 5.4.3).

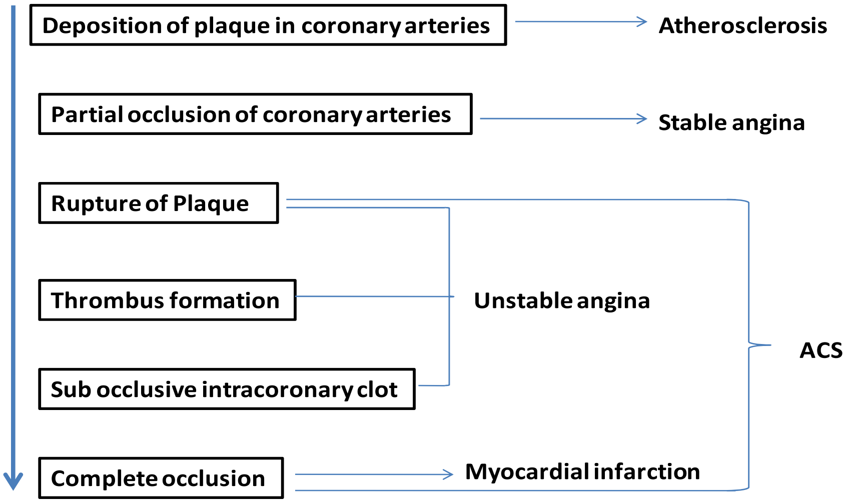

Figure 5.4.3. Spectrum of coronary artery disease

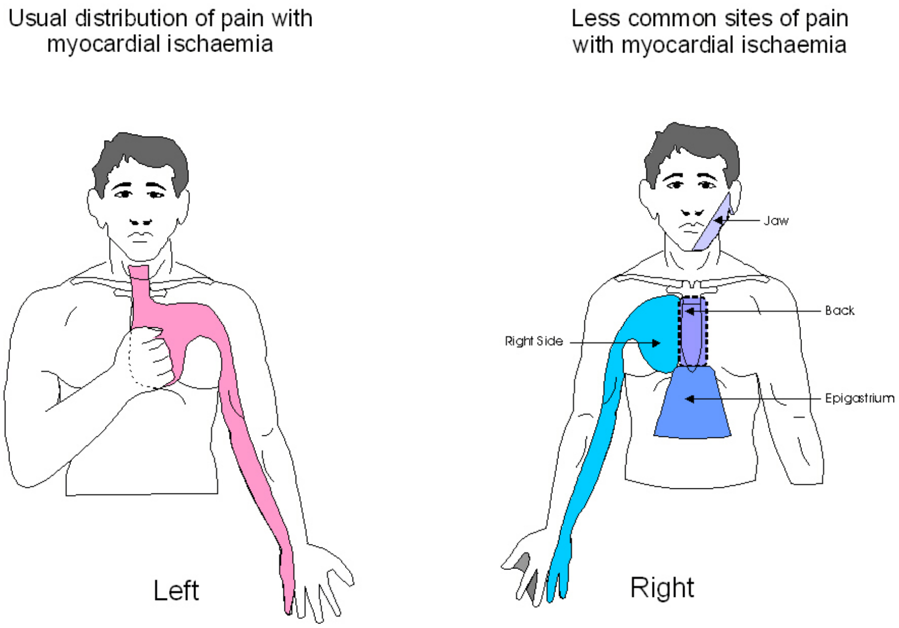

Fig.5.4.4: Figures showing the usual and unusual site of pain of angina

Clinical manifestations:

Chest pain (angina) is the commonest symptom

- **Typical angina**: Substernal pressure radiating to neck, jaw, arm (Fig. 4) with duration <20-30 minutes which may be associated with dyspnea, diaphoresis, palpitations, nausea-vomiting, or lightheadedness; increases with exertion, decreases with rest or NTG. (**Note**: Rest angina is angina occurring at rest and prolonged, usually greater than 20 minutes)
- **MI**: Has increased angina intensity and duration >30 min. Twenty five percent of MIs are clinically silent. Proportion of painless STEMIs is greater in patients with diabetes mellitus and increases with age.

Associated symptoms: Weakness, nausea/vomiting, sweating, apprehension, anxiety, sense of impending doom.

Other presentations, with or without pain

- Sudden-onset breathlessness, loss of consciousness confusional state or sensation of profound weakness
- Rhythm abnormalities or unexplained decrease in arterial pressure
- Evidence of peripheral embolism

Features not characteristics of myocardial ischemia:

- Sharp pain brought by respiratory movement or cough,

- Pain that may be localized by the tip of one finger, particularly over the left ventricular apex or a costochondral junction.
- Very brief episode of pain that lasts a few seconds
- Pain reproduced by movement or palpation over the chest
- Constant pain that lasts for many hours without other ischemic symptoms

Physical examination

- **Focused clinical examinations** for evidence of heart failure, peripheral hypo-perfusion (pallor, diaphoresis, cool extremities), heart murmur, elevated JVP, pulmonary edema should be noted quickly without delaying treatment.

- The presence of severe underlying coronary disease is suggested in patients with clinical **evidence of LV dysfunction, congestive heart failure**

- Pulse rate and blood pressure: Arterial pressure is variable. In most transmural infarctions, systolic pressure decreases by approximately 10–15 mmHg from the preinfarction state.
  - Many patients have normal pulse rate and blood pressure within the first hour of STEMI.
  - Patients with large infarctions have hypotension (systolic blood pressure <100 mmHg and/or sinus tachycardia >100/min)
  - Anterior infarction: About one-fourth of patients have manifestations of sympathetic nervous system hyperactivity (tachycardia and/or hypertension).
  - Inferior infarction: Up to half of patients show evidence of parasympathetic hyperactivity (bradycardia and/or hypotension).
- In right ventricular (RV) infarction, Jugular venous distention is common.

- Look for signs of ventricular dysfunction
  - Third and fourth heart sounds
  - Decreased intensity of the first heart sound
  - Paradoxical splitting of the second heart sound

- Transient mid-systolic or late systolic apical systolic murmur due to dysfunction of the mitral valve apparatus may be present. New, loud (≥Gr 3/6) precordial systolic murmur may be present in ruptured ventricular septum and mitral regurgitation

- Pericardial friction rub in pericarditis (usually develops 24-96 hours after MI)

Electrocardiogram in ACS

- **A 12 lead resting ECG** (± RV3, RV4 for right ventricular MI) should be obtained immediately in patients with ongoing chest pain as rapidly as possible with in 10 minutes of presentation
- A **normal ECG does not exclude** the presence of severe CAD, and should be repeated if strong suspicion in every 4-6 hrs or earlier
- **ECG abnormality** includes:

– Resting **ST segment changes** (depression ≥ 0.5 mm horizontal or down sloping in NSTEACS, convex elevation > 1mm in ≥2 consecutive leads in STEMI, pseudo normalization of ST segment or dynamic changes)

– New pathological **Q-waves** (>0.4 seconds) is considered diagnostic of MI, but may occur with prolonged ischemia

– **T wave**-inversion(≥ 2 mm symmetrical) or a peaked upright T waves may be the first ECG manifestations of Myocardial Ischemia

– Recent onset **LBBB**

– **RVMI** is diagnosed with ST segment elevation in lead V4R, ST elevation in V1 in the presence of ST elevation in inferior leads

– **Non-specific ST and T changes:** ST depression <0.5 mm, T wave inversion <2mm, isoelectric T wave or asymmetric T inversion is less suggestive of myocardial ischemia.

- The **range of normal ST-segment** deviation differs between men and women. ST-elevation (concave upwards) in the V2 or V3 leads of 2·0 mV or less in men and 1·5 mV or less in women, or 1·0 mV or less in other leads, is normal
- ECG **changes that mimic MI** may result from pre-excitation, pericarditis, myocarditis, cardiomyopathy, COPD, pulmonary embolism, cholecystitis, and hyperkalemia; thus the treating physician should be aware.

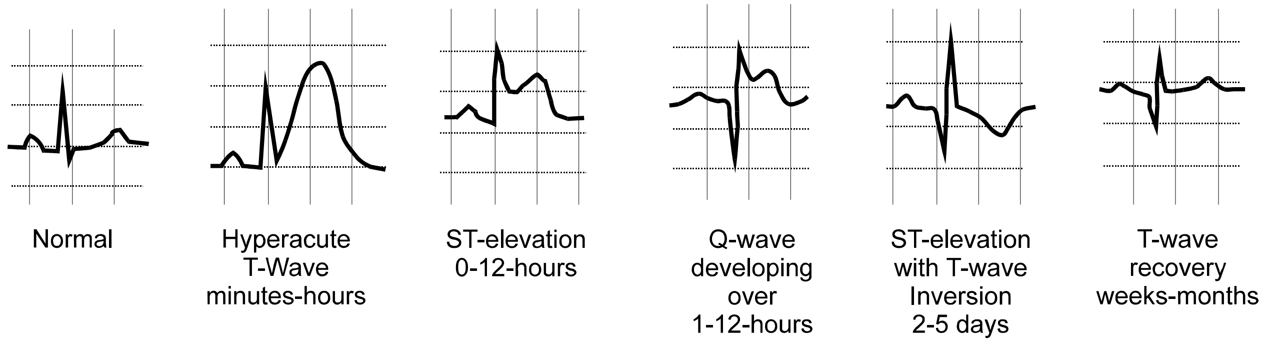

Figure 5.4.5. Evolution of ECG changes in Myocardial infarction

Laboratory studies (depending upon the facilities available):

- **Blood samples** should be sent for cardiac enzymes (biomarkers Troponin I or T and CK-MB)-for diagnosis of ACS; Hemogram, blood urea, creatinine, electrolytes, FBS -for monitoring and Fasting lipid profile- for secondary prevention. Cardiac specific troponin is the preferred biomarker (Table 5.4.1) for diagnosis of STEMI.

Table 5.4.1: Time course of serum markers in acute MI

**Test**	**Onset**	**Peak**	**Duration**
Creatine kinase - total and MB	3-12 hours	18-24 hours	36-48 hours
Troponins	3-12 hours	18-24 hours	Upto 10 days

- A portable **chest radiograph** is useful to exclude other causes of acute chest pain but it should not delay the initiation of therapy

- Imaging: (if facilities are available)

  2D echocardiography and Doppler echocardiography:

Algorithm for the Evaluation and Management of Patients with chest pain

Because symptoms are similar, the differentiation of CSA, UA/NSTEMI and STEMI from that of a non coronary chest pain requires medical evaluation and judgment (figure 5.4.6).

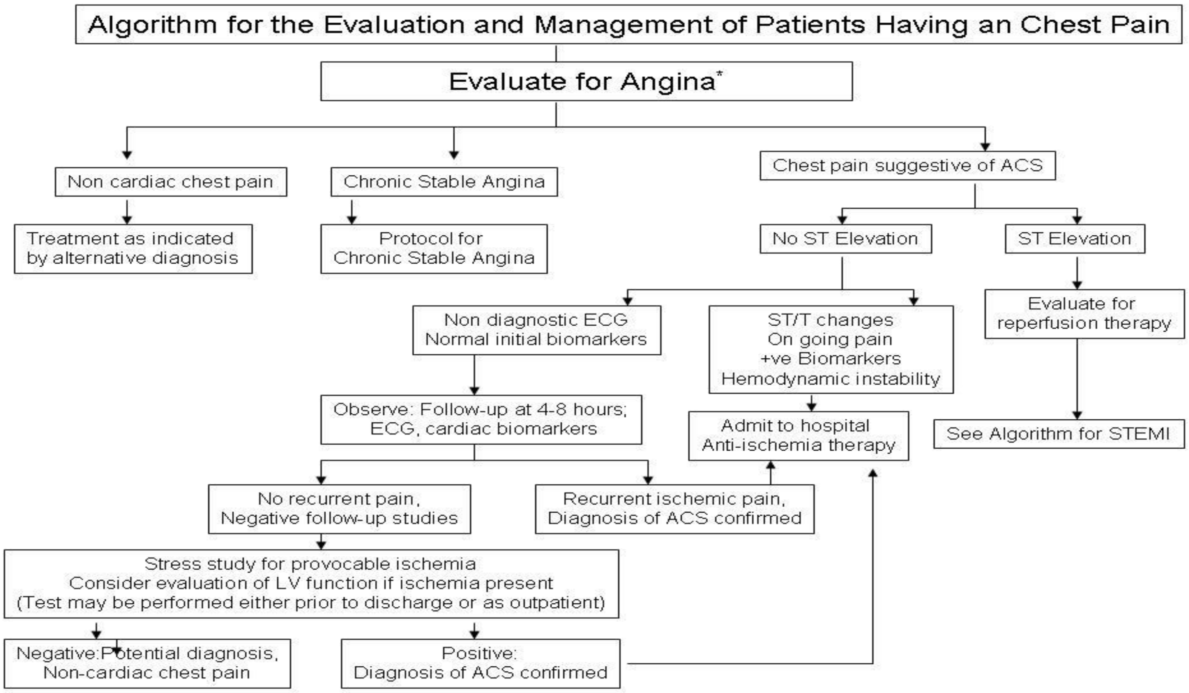

* History, ECG, stress tests

Figure 5.4.6. Algorithm for Evaluation and Management of Patients with Chest Discomfort.

1. Management of Chronic stable angina:

A. History: Clinical Classification of Chest Pain

- Typical angina (**definite** if all 3 present)

1. Retrosternal chest discomfort with a characteristic quality and duration that is
2. Provoked by exertion or emotional stress and
3. Relieved by rest or nitroglycerin

- Atypical angina (**probable**)

Meets 2 of the above characteristics

- Non-cardiac chest pain

Meets ≤1 of the typical angina characteristics

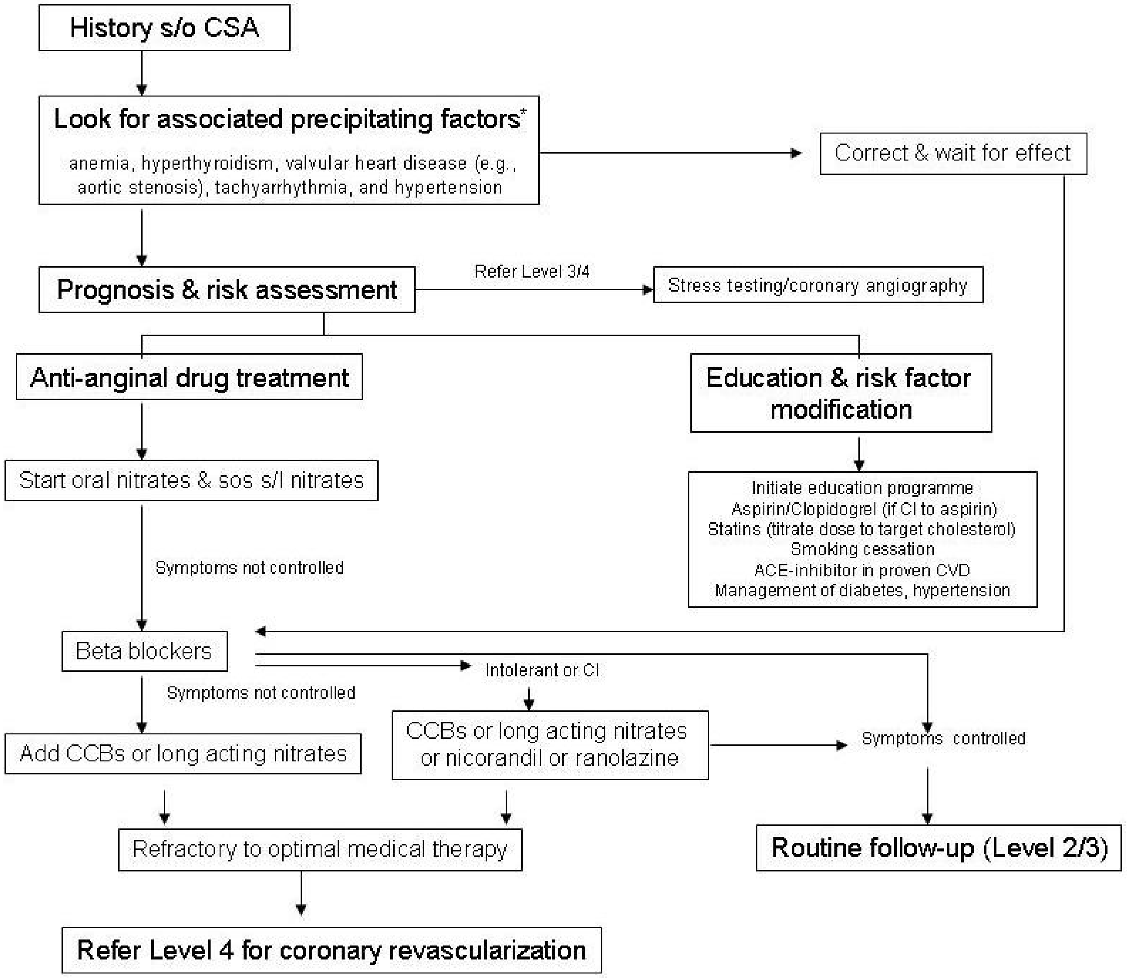

Figure 5.4.7. Management Algorithm of chronic stable angina

Table 5.4.2: Summary of Management of Chronic Stable Angina at Various Level of Health Care

Level 1 (PHC)	Level 2 (CHCs, Sub-divisional hospitals)	Level 3 (District Hospitals)	Level 4 (Medical colleges with facilities for PCI and Tertiary centers)
Diagnose and refer as soon as possible	• Detailed history<br>• Investigation<br>- ECG<br>- Blood sugar, Serum creatinine, Cholesterol<br>- Chest X ray<br>• Treatment<br>- Nitroglycerin (Sublingual)- sos<br>- Oral nitrates<br>- Beta blockers<br>- Aspirin<br>- Statins and ACE inhibitors<br>- Reduce cholesterol < 200 mg/dl<br>- Reduce LDL < 100 mg/ dl<br>• Refer to level 3/ 4, if angina is not controlled despite medication	• Management same as level 2 for new patient<br>• Echocardiography for LV Function	• Management same as level 2 for new patient<br>• Angiography and revascularization if facilities are available for refractory cases

2. Initial management of ACS at emergency department

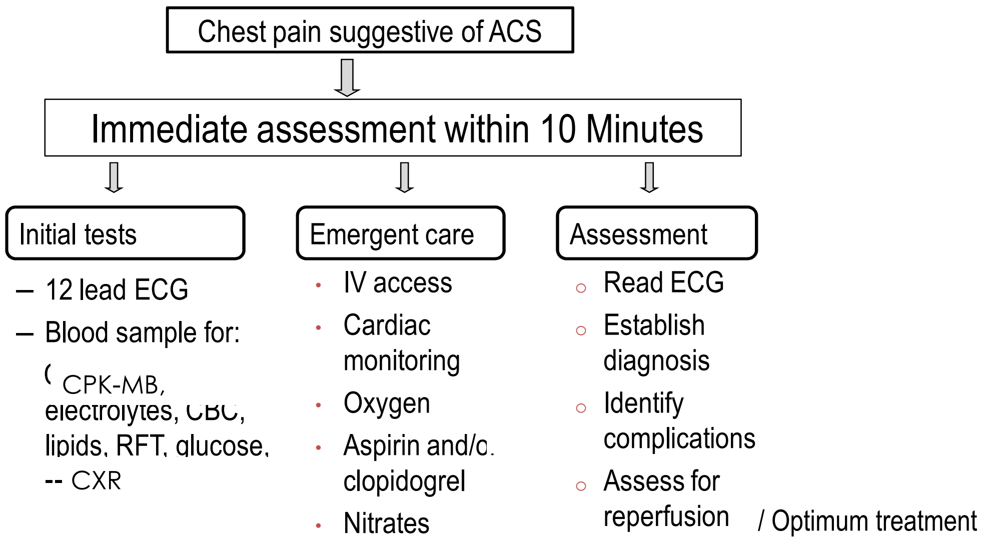

Figure 5.4.8: Flow chart for initial emergency measures in management of ACS

3. Management of Acute Coronary Syndrome (ACS)

Summary of recommendations for management of ACS at different levels				
**Levels of care**	**Level 1 (PHC)**	**Level 2 (CHCs, sub-divisional hospitals)**	**Level 3 (District hospital)**	**Level 4 (Medical colleges with facilities for PCI & Tertiary centers)**
**History**	Chest pain, associated symptoms angina equivalent, Orthopnea, presyncope/syncope	Reassess history	Reassess history	Reassess history
**Examination**	Pulse, BP, Cardiac auscultation, Chest auscultation	Directed physical examination	Directed physical examination	Directed physical examination
**Investigations**	ECG	ECG, Cardiac biomarkers (Trop Tor I / CK-MB), Hemogram, FBS, Lipids, Serum electrolytes & Renal function tests	As for Level 2 and TMT & Echo (if available) - Risk startification for CSA & low risk ACS	As for Level 3 and Cardiac catheterization lab & surgica facilities
**Management**	ECG Aspirin, clopidogrel, s/l nitrate Prompt referral – Level 2 & higher (as per possibility)	Aspirin, clopidogrel, (if not given) s/l nitrate Analgesia-morphine Anti-ischemic therapy (BB, nitrates) ACEI / ARBs if LV dysfuntion Anticoagulant therapy (heparins) as per protocol Statins Thrombolysis for STEMI Refer – Level 3 for further evaluation of low risk ACS Level 4 for Primary PCI in case of CI to thrombolysis or Rescue PCI for failed lysis, Early intervention for high risk ACS Counselling & health education	Treatment protocol as for Level 2 and Thrombolysis for STEMI Refer – Level 4 for Primary PCI in case of CI to thrombolysis or Rescue PCI for failed lysis, Early intervention for high risk ACS Counselling & health education	State of the art management, including, Primary & rescue PCI.

* At sub-centre: give Tab. Aspirin 300mg stat with prompt referral to nearest Level 1 care
**Markers of successful lysis: decrease in chest pain, ST resolution of 50% or more and the development of a terminal negative T wave in the lead with the highest ST elevation

4. Medication Dosing and Administration:

Aspirin
• 300 mg chewed and swallowed (150 mg × 2) upon presentation, then 150 mg daily indefinitely.

Clopidogrel
• 300-mg oral loading dose, then 75 mg PO daily for 9 to 12 mo.

Heparin
• LMWH (Enoxaparin1 mg/kg SC Q12 h or Dalteparin 120 IU/kg SC (max 10,000 IU) Q12h or, until PCI or till hospital admission)

ß-Blockers (should be initiated in first 24 hours if no contraindications in small doses)
• Oral Metoprolol 25-50 mg PO BD.
• Carvedilol 6.25-25mg BD. (if LV dysfunction)
• Patient with early contraindication should be reevaluated for b-blocker therapy for secondary prevention

Nitroglycerin
• 0.4 mg sublingual Q 5 min × 3 for persistent ischemic pain or IV infusion starting at 5-10 μg/min with up titration for persistent ischemic pain. Oral long acting nitrates once/twice daily.

Morphine sulfate
• 2–4 mg IV every 5–10 minutes until pain is relieved or side effects develop
•Side effects: Nausea, vomiting, respiratory depression and hypotension .

Oxygen
• 2–4 L/min by nasal cannula to maintain oxygen saturation > 90%

ACE inhibitors
• Captopril 6.25 mg TID, titrate up as tolerated
- Ramipril 2.5-5mg BD
- ARBs, (Valsartan 20-160 mg BD) in patients intolerant to ACE inhibitors with evidence of LV dysfunction.
- **A**ldosterone blockers (spironolactone 25mg OD, eplerenone 25-50 mg OD)
  - Post-STEMI patients who meets the following
    - No significant renal failure (Cr < 2.5 men or 2.0 for women)
    - No hyperkalemia > 5.0
    - LVEF < 40%
    - Symptomatic Congestive heart failure or Diabetes Mellitus

Insulin consider insulin infusion in first 48 hours to normalize blood glucose

5.5. PREVENTION AND MANAGEMENT OF STROKE

What is a Stroke?

A stroke means that part of the brain is suddenly damaged. If an artery in the brain becomes blocked by a thrombus, it causes a stroke. If an artery in the brain leaks then too it damages the brain and causes a stroke.

Atheroma is also known as 'atherosclerosis' or 'hardening of the arteries'. Patches of atheroma are also called 'plaques' of atheroma. Patches of atheroma are like small fatty lumps that develop within the inside lining of arteries (blood vessels).The thrombus usually forms over some atheroma.

A temporary lack of blood supply to a part of the brain is known as TIA or transient ischemic attack.

Prevent Strokes

Stroke can be prevented by controlling high blood pressure, avoiding tobacco use and leading a healthy life style. Simple tips include the following:

- Keeping blood pressure well under control.
- In case of positive family history of stroke yearly evaluation of risk factors such as hypertension and diabetes.
- Eating healthy (plenty of whole grains, fruits and vegetables in the daily diet).
- Avoiding refined flours, sugars and foods rich in trans fat such as biscuits, deep fried foods etc.

**Box 5.5.1: Practical advice to patients on how strokes can be prevented**	
⇒	Know the **blood pressure** . Have the blood pressure checked at least once a year, and, if it is elevated, treat it diligently, to keep it under control.
⇒	Stopping the use of both smoking and non-smoking forms of tobacco.
⇒	It is preferable to avoid alcohol due to several other ill effects on health. However for individuals who consume alcohol the consumption should be moderate (daily consumption of not more than a glass of wine or 30-50ml of hard drinks such as whisky, brandy or similar products with high alcohol content).<br>Avoid binge drinking. It is a major risk factor for stroke because it can acutely elevate blood pressure.
⇒	Including exercise in the daily routine

⇒	Consuming a low-salt, low-fat diet
⇒	Preferring whole grains and whole pulses and eating 10-40 gms of unsalted non-fried nuts everyday.
⇒	**Identifying circulatory problems that could increase the risk of stroke and Atrial fibrillation**
⇒	Screening for hypercholesterolemia. If more than 200mg/dl, lower it by lifestyle changes like regular exercise and change in diet along with statins.
⇒	Controlling **diabetes**, if present concurrently.
⇒	**Avoiding deep vein thrombosis. If a patient is recovering from illness or a surgery and is in bed, then make sure he/she exercises the legs by raising it up and down 10-15 times every day.**

Educate the patients that if there are stroke symptoms, including sudden weakness of the face or a limb, a blurring of vision, dizziness, or an intense headache, he/she should seek immediate medical attention

Management

Patients of stroke presenting within 6 hours of onset of symptoms should be referred to a secondary care for initial assessment and management. The follow-up of patients presenting with a completed stroke not requiring acute care (such as respiratory distress) can be managed at the PHC level.

Identification of an acute event

- Sudden onset of weakness of one half of body or one part of body
- Sudden onset of inability or difficulty in speech
- Sudden onset of imbalance
- Sudden onset of blindness
- Sudden onset of dizziness or spinning
- Sudden severe headache
- Seizures
- Sudden loss of consciousness

All patients with above symptoms should be examined by qualified medical practitioners

Guidelines for stroke treatment at a secondary health care level

1. If available a plain CT scan should be done in all cases; contrast if indicated.

2. Secure the airway by keeping the patients head to a side; if breathing is compromised assisted ventilation to be provided and circulation should be maintained by securing a good IV line and infusing 5% dextrose.
3. Elevated blood pressure should be managed by nitroprusside, labetalol (under monitoring) or captopril in titrated doses. ***In most places only captopril will be available and this can be given sublingually too.*** The BP should not be brought down rapidly. The systolic should be around 140 mmHg and diastolic between 80-90 mmHg.
4. Avoid cerebral decongestants (mannitol, glycerol) unless there is evidence of raised Intra-Cranial-Pressure with signs of decerebration.
5. Provide supportive care to prevent deep vein thrombosis by prophylaxis with 5000 units heparin BD (or equivalent units of low molecular weight heparin if available and affordable).
6. Acute rehabilitation includes proper positioning, dysphasia management, passive movements of limbs, bowel/bladder care with active involvement of family members

Guidelines for referral to a tertiary health care level

- If CT shows significant pressure effect, or middle cerebral artery (MCA) dense sign suggesting massive infarction, refer to tertiary centre
- If CT shows intra-parenchymal hemorrhage with midline compression, or a cerebellar infarct or hemorrhage refer to higher centre.
- If CT shows primary subarachnoid hemorrhage refer to higher centre

Guidelines for follow-up of stroke at all levels

- First follow up at 2 weeks. Follow up to be kept at 3 or 6 monthly intervals depending on individual merits of the case.
- Look for Functional recovery,
- Check blood pressure and blood sugars,
- Monitor compliance with rehabilitation measures.
- Continue Aspirin. Use of clopidogrel or combination antiplatelet agents such as low dose aspirin plus extended release dypiridamole to be instituted on individual merits of the case after risk stratification at the secondary or tertiary care centers. Cardio-embolic strokes will need oral anticoagulants with monitoring of prothrombin time.
- Dietary and lifestyle modification. Counseling regarding vocational guidance and eventual return to work

Guidelines for stroke prevention:

1. **Identification of those at high risk**

- High blood pressure ,
- History of heart disease,
- History of TIA,
- Past history of stroke
- Diabetes,
- Tobacco users,
- Family h/o increased risk for vascular disease,
- Obesity- sedentary life style ,
- On oral contraceptives,
- Hyperlipidemia,
- Family history of stroke

Individuals belonging to the above category are at a higher risk for stroke. Such individuals have to undertake preventive measures for stroke. Refer the section on 'Therapeutic lifestyle management' for lifestyle changes. Subjects with established vascular diseases have to take extra precautions as described below.

2. Primary prevention in high risk individuals

Advise to adopt a healthy life style and please refer to section on healthy life style.

SUGGESTED READING:

- World Health Organization. Preventing chronic diseases: a vital investment. Geneva, 2005.

- Reddy KS, Shah B, Varghese C, Ramadoss A. Responding to the threat of chronic diseases in India. The Lancet 2005; 366(3498):1744-1749.

- Mohan V, Sandeep S, Deepa R, Shah B, Varghese C. Epidemiology of type 2 diabetes: Indian scenario. Indian J Med Res 2007; 125(3):217-230.

- Report of the Joint International Society and Federation of Cardiology/World Health Organization Task Force on Standardization of Clinical Nomenclature. Nomenclature and criteria for diagnosis of ischemic heart disease. Circulation 1979; 59: 607–9.

- World Health Organization. Prevention of Cardiovascular disease; Pocket guidelines for assessment and management of cardiovascular risk. Geneva 2007.

- Reddy KS, Naik N, Prabhakaran D. Hypertension in the developing world: a consequence of progress. Curr Cardiol Rep 2006; 8(6):399-404.

- Guidelines for prevention of Ischemic Heart Disease in India by cardiology society of India.2003.

- World Health Organization. WHO CVD risk assessment and management for low and medium resource settings. Geneva, 2002.

- Integrated management of cardiovascular risk. A report of WHO meeting 9th – 10th July. Geneva. (Link: http://www.who.int/cardiovascular_diseases/media/en/635.pdf)

- World Health Organization. Prevention of recurrent heart attacks and strokes in low and middle income populations. Evidence based recommendations for policy makers and health professionals. Geneva, 2003.

- World Health Organization. Avoiding heart attacks and strokes. Don't be a victim protect yourself. Geneva 2005.

- Thygesen, K, Alpert, JS, White, HD, et al. Universal definition of myocardial infarction: Kristian Thygesen, Joseph S. Alpert and Harvey D. White on behalf of the Joint ESC/ACCF/AHA/WHF Task Force for the Redefinition of Myocardial Infarction. Eur Heart J 2007; 28:2525.

- White HD. Evolution of the definition of myocardial infarction: what are the implications of a new universal definition? Heart 2008; 94: 679–84.

LIST OF PARTICIPANTS/ EXPERTS IN FINALIZATION WORKSHOP:

Venue: Dr RML PGIMER, New Delhi

1. **DIRECTORATE GENERAL OF HEALTH SERVICES**
    a. **Dr R K Srivastava, DGHS**
    b. **Dr D C Jain, DDG (LME)**
    c. **Dr W D Bhutia, CMO (WDB)**
    d. **Dr Sudhir Gupta, CMO (NCD)**
    e. **Dr Ritu Rana, Consultant-NCD**
2. **PGIMER, CHANDIGARH**
    a. **Prof. K K Talwar, Director**
    b. **Dr Shiv Bagga, Assistant Professor, Cardiology**
    c. **Dr Binod Patro, Assistant Professor, School of Public Health**
3. **Dr RML PGIMER, NEW DELHI**
    a. **Dr Harsh Wardhan, Head, Cardiology**
    b. **Dr Ranjit Nath, Assistant Professor, Cardiology**
4. **Center for Chronic Disease Control, New Delhi**
    a. **Dr D Prabhakaran, Executive Director**
5. **WHO – India**
    a. **Dr J S Thakur, Cluster Focal Point, NDS Cluster**
    b. **Dr Sitanshu Kar, National Consultant, NDS Cluster**
